# Heat stress leads to rapid lipid remodeling and transcriptional adaptations in *Nicotiana tabacum* pollen tubes

**DOI:** 10.1093/plphys/kiac127

**Published:** 2022-03-18

**Authors:** Hannah Elisa Krawczyk, Alexander Helmut Rotsch, Cornelia Herrfurth, Patricia Scholz, Orr Shomroni, Gabriela Salinas-Riester, Ivo Feussner, Till Ischebeck

**Affiliations:** 1 Department of Plant Biochemistry, Albrecht-von-Haller-Institute for Plant Sciences and Göttingen Center for Molecular Biosciences (GZMB), University of Göttingen, Göttingen 37077, Germany; 2 Service Unit for Metabolomics and Lipidomics, Göttingen Center for Molecular Biosciences (GZMB), University of Göttingen, Göttingen 37077, Germany; 3 NGS—Integrative Genomics Core Unit (NIG), University Medical Center Göttingen (UMG), Institute of Human Genetics, Göttingen 37077, Germany; 4 Institute of Plant Biology and Biotechnology (IBBP), University of Münster, Green Biotechnology, Münster 48143, Germany

## Abstract

After reaching the stigma, pollen grains germinate and form a pollen tube that transports the sperm cells to the ovule. Due to selection pressure between pollen tubes, pollen grains likely evolved mechanisms to quickly adapt to temperature changes to sustain elongation at the highest possible rate. We investigated these adaptions in tobacco (*Nicotiana tabacum*) pollen tubes grown in vitro under 22°C and 37°C by a multi-omics approach including lipidomic, metabolomic, and transcriptomic analysis. Both glycerophospholipids and galactoglycerolipids increased in saturated acyl chains under heat stress (HS), while triacylglycerols (TGs) changed less in respect to desaturation but increased in abundance. Free sterol composition was altered, and sterol ester levels decreased. The levels of sterylglycosides and several sphingolipid classes and species were augmented. Most amino acid levels increased during HS, including the noncodogenic amino acids γ-amino butyrate and pipecolate. Furthermore, the sugars sedoheptulose and sucrose showed higher levels. Also, the transcriptome underwent pronounced changes with 1,570 of 24,013 genes being differentially upregulated and 813 being downregulated. Transcripts coding for heat shock proteins and many transcriptional regulators were most strongly upregulated but also transcripts that have so far not been linked to HS. Transcripts involved in TG synthesis increased, while the modulation of acyl chain desaturation seemed not to be transcriptionally controlled, indicating other means of regulation. In conclusion, we show that tobacco pollen tubes are able to rapidly remodel their lipidome under HS likely by post-transcriptional and/or post-translational regulation.

## Teaser

*Nicotiana tabacum* pollen tubes react to heat stress with metabolomic adaptations, transcriptional adjustments, and rapid and reversible lipid remodeling.

## Introduction

Around 80% of the >350,000 known plant species are flowering plants (angiosperms) that mainly rely on sexual reproduction to pass on their genes to the next generation (Christenhusz and Byng, 2016). Sexual reproduction is also the prerequisite for seed and fruit set in crop plants. In most angiosperms, the male gametophyte—the pollen containing the sperm cells or the generative cell that later divides into the two sperm cells—double-fertilizes the female gametophyte. To this end, the pollen has to land on the stigma of a pistil and adhere, rehydrate, and germinate there. Since seed plant sperm cells are immotile, they have to be delivered through the female reproductive tissues to the ovule by tip-growing pollen tubes that are formed by the vegetative cells of the pollen (Sprunck, 2020). In some species, pollen tubes have to grow several centimeters to reach the egg-cell containing ovule. Maize (*Zea mays*) pollen tubes, for example, can grow up to 50 cm in length with a speed of more than 1 cm per hour (Mascarenshas, 1993). Especially for these very long pollen tubes, it is unlikely that enough lipids for the elongation are stored in the pollen grain, but the tubes rather depend on high de novo synthesis for their growth (Ischebeck, 2016). This assumption is supported by the fact that proteins involved in fatty acid synthesis are 5–10 times more abundant in maturing pollen and growing pollen tubes of tobacco (*Nicotiana tabacum*) than in leaves or roots (Ischebeck et al., 2014; Ischebeck, 2016).

The process of sexual reproduction in plants is very sensitive to abiotic stresses like heat stress (HS) or cold stress. Male gametophytes are especially sensitive to HS during all stages of their life and are generally more susceptible than female tissues (Hedhly, 2011; Santiago and Sharkey, 2019). One effect of HS on developing pollen is early tapetum degradation likely due to accumulation of reactive oxygen species (ROS) and the consequent disruption of concerted ROS signals (Zhao et al., 2018). Furthermore, anthers can fail to release pollen grains, possibly due to heat-induced changes in cell wall composition, sucrose transport, and water movement (Santiago and Sharkey, 2019). Also, the developing male gametophyte itself can encounter problems at different stages of development (Müller and Rieu, 2016; Raja et al., 2019; Santiago and Sharkey, 2019).

The effects of HS on pollen germination and pollen tube growth are less well studied than those on pollen development but have also been addressed in several studies. It was shown that tomato (*Solanum lycopersicum*) pollen germination is negatively affected by HS in in vitro experiments (germination and pollen tube growth in medium outside of female tissues); an effect also attributed to elevated ROS levels (Luria et al., 2019). Various sorghum (*Sorghum bicolor*) genotypes show in vitro pollen germination reductions of 2%–95% under different HSs (Sunoj et al., 2017). In vivo experiments with pollen from rice (*Oryza sativa*) or pinyon pine (*Pinus edulis*) gave similar results (Flores-Rentería et al., 2018; Shi et al., 2018).

Inhibited pollen tube growth has been observed as well. Experiments on in vivo grown cotton (*Gossypium hirsutum*) pollen tubes attribute reduced pollen tube growth to HS-induced reduction of soluble carbohydrate content in the pistil (Snider et al., 2011a, 2011b). Heat-induced pollen tube growth inhibition in rice was described to be likely due to altered auxin homeostasis within the pistil (Zhang et al., 2018). These experiments highlight the importance of biochemical interactions between pollen tubes and the surrounding pistil tissue during stress. However, pollen tube growth inhibition is also observed in vitro. Experiments with tomato pollen showed maximal germination rates at 15°C and maximal pollen tube lengths at 25°C, higher (or lower) temperatures were inhibitory (Karapanos et al., 2010). Another in vitro study in tomato showed that high temperature-induced ROS inhibit pollen tube growth and that elevated flavonol levels can counteract the heat-induced ROS imbalance (Muhlemannet al., 2018). Also, in vitro pollen tube growth of cotton, rice, and Arabidopsis (*Arabidopsis thaliana*) is inhibited by high temperatures (Boavida and McCormick, 2007; Song et al., 2015; Coast et al., 2016), indicating that growth inhibitions are not solely due to altered crosstalk with the female tissue, but also due to effects in the pollen tube itself.

A challenge all organs of plants have to meet during elevated temperatures is maintaining membrane fluidity and integrity; this holds true for the plasma membrane as well as for intracellular membranes (Niu and Xiang, 2018). Among the challenges, plant membranes have to meet under elevated temperatures are the prevention of bilayer disintegration (because of membrane hyperfluidity under high temperatures) and peroxidation of unsaturated fatty acids by ROS (Higashi and Saito, 2019).

The effects of long- and short-term HS on the leaf lipidome of different species have been reviewed recently (Higashi and Saito, 2019; Lu et al., 2020): levels of glycerophospholipids containing saturated or mono-unsaturated acyl chains increase; while most glycerophospholipid species with polyunsaturated acyl chains decrease. At the same time, triacylglycerol (TG) levels increase.

*Nicotiana tabacum* used in this study is a versatile model organism that is also very suited to study pollen tube growth. Here, we show that tobacco pollen tubes can grow well at a relatively high temperature of 37°C and monitor the adaptions of the pollen tubes on its lipidome, transcriptome, and metabolome.

## Results

### Tobacco in vitro pollen tube growth is not inhibited by elevated temperatures

**Figure 1:**
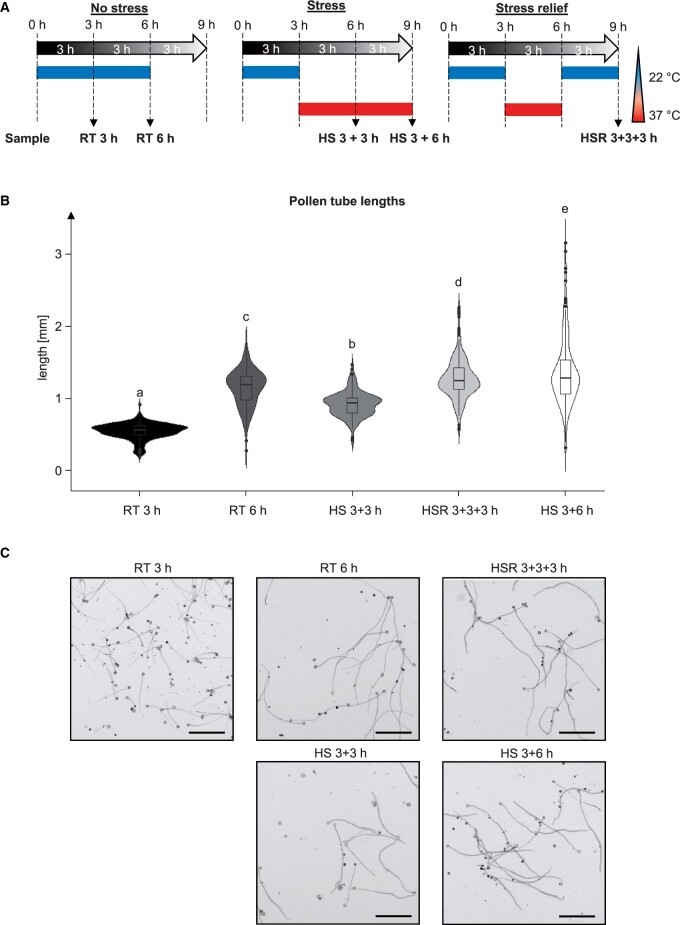
Pollen tubes were subject to different temperature regimes. A, Schematic depiction of the experimental setup. Pollen tubes were grown under five different conditions: either unstressed for 3 or 6 h at RT (22°C; 3 h, 6 h); at RT for 3 h followed by either 3 or 6 h of HS (37°C; 3 + 3 h, 3 + 6 h); or for 3 h at RT, followed by 3 h HS at 37°C and then another 3 h at RT to assay HSR (3 + 3 + 3 h). B, Violin plot of measured pollen tube lengths under the different conditions. Boxplots are shown within the diagram (center line corresponds to median, upper and lower hinge correspond to 25th and 75th percentiles, upper and lower whisker extend to largest/smallest value within 1.5× inter-quartile range of the respective hinge, and outliers beyond the whiskers are presented individually). ANOVA was performed (significance level *α* = 0.05), followed by post hoc Tukey’s analysis. Results are presented as compact letter display of all pair-wise comparisons. *n* = 183–201. C, Images of pollen tubes grown under the indicated conditions. Bars = 0.5 mm.

The *N.* *tabacum* genome has been sequenced; tobacco flowers are large and rich in pollen that germinates and grows tubes effectively in vitro (Read et al., 1993; Edwards et al., 2017) making it a suitable species to use in a study on pollen tubes. First, we tested if tobacco pollen tubes also grow at higher temperatures and thereby pose a possible model to study successful heat adaptation. For this, we analyzed pollen tube elongation under five different conditions (Figure 1A) with or without HS: 3 h of growth at room temperature (RT; 22°C, 3 h), 6 h of growth at RT (6 h), 3 h growth at RT and then 3 h growth under HS (HS 3 + 3 h), 3 h growth at RT and then 6 h growth under HS (HS 3 + 6 h), and 3 h growth at RT followed by 3 h growth under HS, and finally 3 h at RT for HS relief (HSR; 3 + 3 + 3 h). The chosen time points do not allow a comparison of extended HS and continuous growth at RT, but a comparison between extended HS and HSR can be drawn. Pollen tubes grown at RT for 3 h reached an average length of 0.46 mm and after 6 h they more than doubled their lengths, reaching 1.1 mm (Figure 1, B and C). If shifted to HS after 3 h of RT growth, pollen tube length after 6 h only reached 0.83 mm. This is a growth reduction of around 38% in comparison to RT 6 h, indicating that while pollen tubes can still grow at this temperature their growth is hampered. If subjected to prolonged HS of 6 h, tubes averaged a length of 1.27 mm while HS relieved pollen tubes grew to 1.19 mm.

### Relative glycerophospholipid and galactoglycerolipid composition changes under HS

HS usually induces membrane remodeling in order to maintain membrane integrity. Sustaining pollen tube growth under elevated temperatures thus likely requires lipidomic adaptations, too. To assay such a remodeling, pollen tubes were grown in five replicates under the described temperature regimes (Figure 1A). Lipids were extracted from the tubes and attached medium. Then, glycerolipids (including neutral glycerolipids and galactoglycerolipids), glycerophospholipids, sphingolipids, and sterol lipids (sterol conjugates) were analyzed by ultra-performance liquid chromatography (UPLC)-nanoelectrospray ionization (nanoESI)-tandem mass spectrometry (MS/MS); free sterols were analyzed by gas chromatography–mass spectrometry (GC–MS). In total, 383 molecular species from 26 lipid subclasses were detected and relative lipid subclass-specific profiles were calculated as percent values of the individual subclasses. Abundances of these subclasses were expressed by normalization of the absolute peak area to the values after 3 h of RT growth (Supplemental Datasets 1–S11). Digalactosylmonoacylglycerol, monogalactosylmonoacylglycerol, sulfoquinovosylmonacylglycerol, sulfoquinovosyldiacylglycerol, phosphatidylinositol (PI)-monophosphate (PIP), PI-bisphosphate (PIP_2_), and lysophosphatidylserine could not be detected in any of the samples. Ceramide-phosphate (CerP), glucuronosylinositolphosphoceramide (GlcA-IPC), hexosyl-GlcA-IPC, and hexosylhexosyl-GlcA-IPC were also not found.

**Figure 2:**
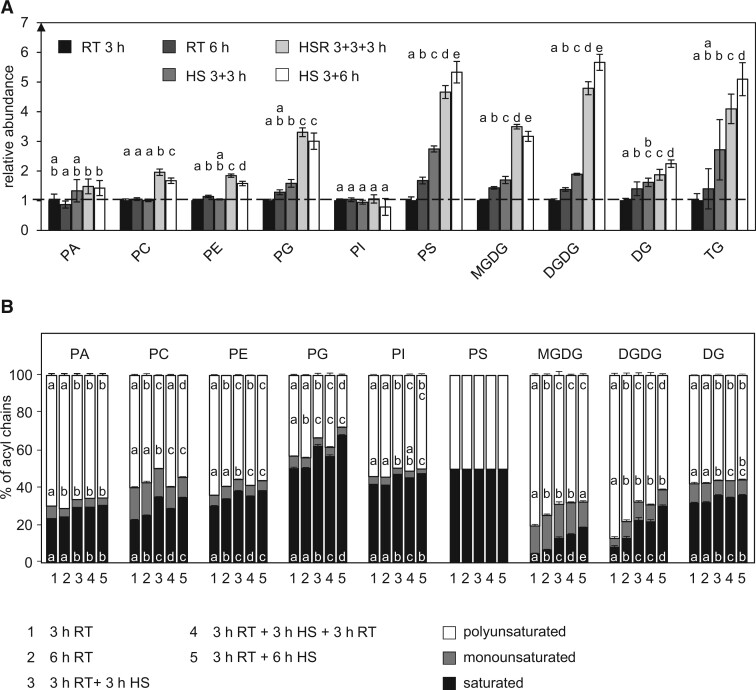
HS changes lipid subclass composition and decreases saturation of acyl chains A, Abundances of the depicted lipid subclasses of pollen tubes grown under different temperature regimes were measured by UPLC-nanoESI-MS/MS and normalized to their respective values after 3 h RT. B, Relative saturation of the respective lipid subclasses under the same temperature regimes. The levels of the molecular lipid species containing zero (saturated), one (monounsaturated), or more than one (polyunsaturated) double bonds in their fatty acid residues were summed per lipid subclass and converted into relative molar proportions. *n* = 5. Error bars represent standard deviation. For statistical analysis, ANOVA was performed (significance level *α* = 0.05), followed by post hoc Tukey’s analysis. Results are presented as compact letter display of all pair-wise comparisons.

HS had only mild effects on the relative abundances of most membrane lipid classes (Figure 2A). For phosphatidic acid (PA), phosphatidylcholine (PC), PI, and phosphatidylethanolamine (PE), only subtle or no changes were observed when the tubes were grown for an additional 3 h at RT or 3 h under HS (total growth of 6 h). After a total growth of 9 h, the relative amounts of PC and PE increased, but their relative accumulation was slightly reduced in heat-stressed tubes when compared with stress-relieved tubes. Stronger relative increases were observed for the other membrane lipids, especially so after prolonged pollen tube growth. Particularly digalactosyldiacylglycerol (DGDG), phosphatidylserine (PS), and TG showed strong relative increases upon heat treatment (Figure 2A). The effects on the synthesis and breakdown of diacylglycerol (DG), an intermediate of several lipid pathways, were less pronounced (Figure 2A).

Looking at overall saturation levels, the relative proportion of saturated acyl chains increased in all membrane glycerolipid subclasses except for PS and DG, while the relative abundance of polyunsaturated acyl chains decreased (Figure 2B). Shifting the tubes back to RT partly led to a reversion of the effects in PC, PE, and phosphatidylglycerol (PG) while relative levels in monogalactosyldiacylglycerol (MGDG) and DGDG remained approximately constant.

**Figure 3:**
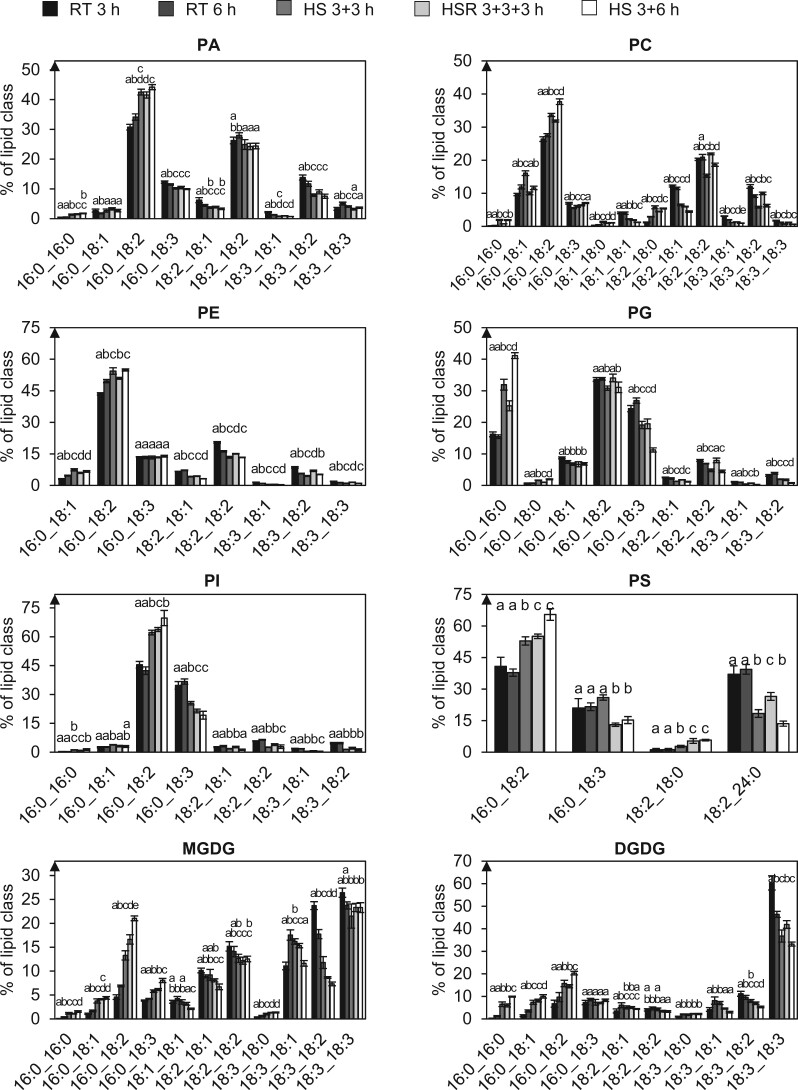
Glycerophospholipid and galactoglycerolipid species with saturated acyl chains increase under HS. The depicted lipid subclasses from pollen tubes grown under different temperature regimes were investigated. Lipid subclass profiles were obtained by UPLC-nanoESI-MS/MS. Values are given as mol % of all detected lipid species per subclass, only major lipid species (>1% of total) are depicted. *n* = 5. Error bars represent standard deviation. For statistical analysis, ANOVA was performed (significance level *α* = 0.05), followed by post hoc Tukey’s analysis. Results are presented as compact letter display of all pair-wise comparisons.

Regarding the acyl chain composition (Figure 3), a relative increase in most 16:0- and 18:0-containing glycerophospholipid species was observed after 3 h of HS, while species without saturated acyl chains generally decreased in relative abundance. If pollen tubes were shifted back to RT, most species containing 16:0 and 18:0 decreased again; while lipids containing no saturated acyl chains increased.

**Figure 4:**
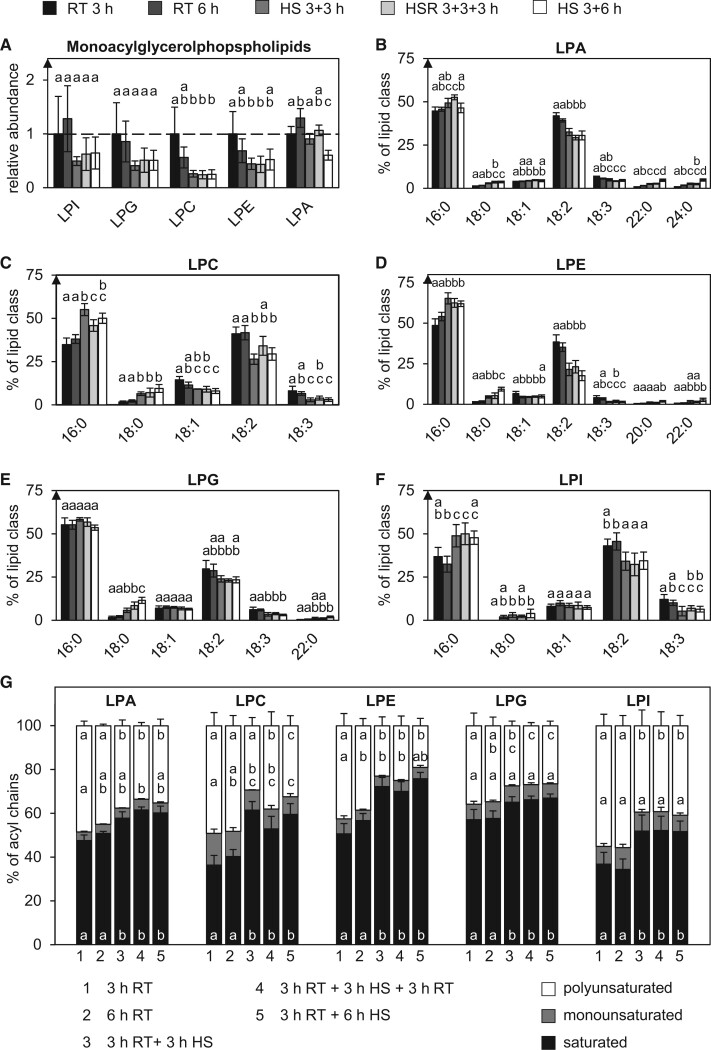
Monoacylglycerphospholipid species with saturated acyl chains increase under HS. The depicted lipid subclasses from pollen tubes grown under different temperature regimes were investigated. A, Relative abundances of the lipid subclasses were measured by UPLC-nanoESI-MS/MS and normalized to their respective values after 3 h RT. B–F, Molecular species within the individual subclasses are given as mol % of all detected lipid species per subclass. Only major lipid species (>1% of total) are depicted. G, Relative saturation of the respective lipid classes under the same temperature regimes. The abundance of the molecular lipid species containing zero (saturated), one (monounsaturated), or more than one (polyunsaturated) double bonds in their acyl residues were summed per lipid subclass and converted into relative proportions. *n* = 5. Error bars represent standard deviation. For statistical analysis, ANOVA was performed (significance level *α* = 0.05), followed by post hoc Tukey’s analysis. Results are presented as compact letter display of all pair-wise comparisons.

Monoacylglycerophospholipids that might negatively affect membrane rigidity (Henriksen et al., 2010) were strongly reduced under HS (Figure 4A). The relative proportion of most monoacylglycerophospholipids containing 16:0 also increased after 3 h of HS (Figure 4, B–F). The relative amounts of 18:2- and 18:3-containing species on the other hand declined leading to a higher saturation (Figure 4G).

The general trend observed in the galactoglycerolipids MGDG and DGDG is the same as for glycerophospholipids: 16:0-containing lipid species increased, while 18:3-containing lipid species decreased. Stress relief partially reversed the observed effects again for some species but not for all (Figure 3).

**Figure 5:**
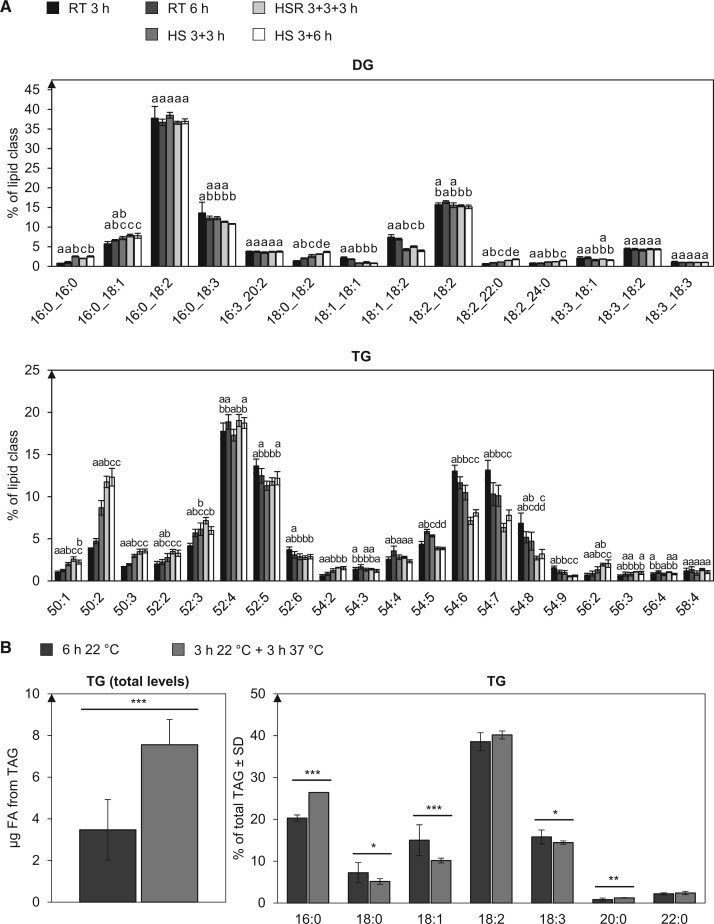
TG accumulates under HS and becomes more saturated. A, The depicted lipid subclasses from pollen tubes grown under different temperature regimes were investigated. Lipid subclass profiles were measured by UPLC-nanoESI-MS/MS. Values are given as mol % of all detected lipid species per subclass. Only major lipid species (>1% of total) are depicted. For statistical analysis, ANOVA was performed (significance level *α* = 0.05), followed by post hoc Tukey’s analysis. *n* = 5. Error bars represent standard deviation. Results are presented as compact letter display of all pair-wise comparisons. B, Absolute quantification of TG from heat stressed and non-stressed pollen tubes per 1 mg of dry pollen. Also shown is the relative contribution of the respective fatty acids to the total TG amount. Measurements were performed with GC-FID and quantified as peak areas. *n* = 5. Error bars represent standard deviation. ***P < 0.005; **P < 0.001; *P < 0.05; determined by Student’s *t* test.

Fewer changes were observed in the acyl chain composition of the glycerolipids DG and TG (Figure 5A). DG profiles remained more or less constant under all tested conditions. For TG, some changes over time but almost no changes in response to HS were observed.

To further validate the relative increase of TG levels under HS (Figure 2A), and to get information on absolute TG levels, TG content was also quantified by gas chromatography-flame ionization detection (GC-FID) (Figure 5B). Heat-stressed pollen tubes (3 h RT + 3 h HS) produced more than twice the absolute amount of TG as control pollen tubes (6 h RT) did.

### Pollen tube sphingolipid abundance increases under HS

**Figure 6:**
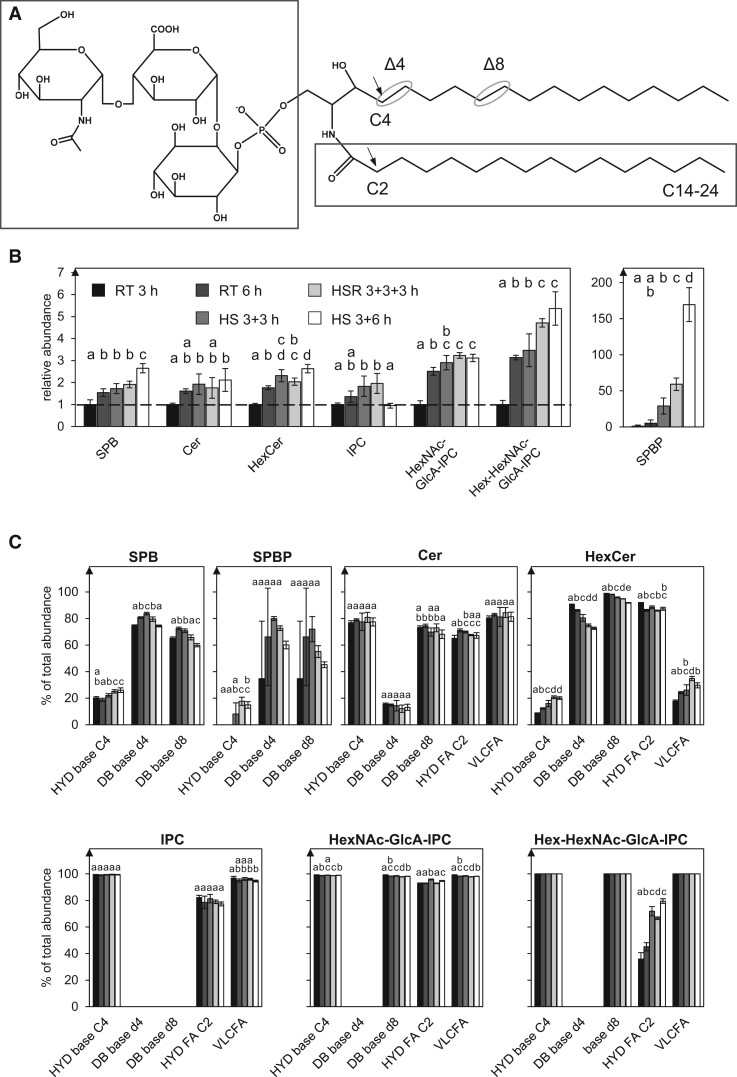
Sphingolipid composition and modifications are affected by HS. A, Sphingolipids consist of a SPB bound to an acyl chain of variable length (right box) forming a ceramide. They can carry a head group (left box) consisting of a hexose (HexCer), a phosphate (e.g. SPBP), a phosphate fused to inositol (IPC) that can be further linked via GlcA to additional sugars as depicted for a HexNAc-GlcA-IPC that can also be linked to a further hexose (Hex-HexNAc-GlcA-IPC). In addition, sphingolipids can be modified at the SPB by a double bond in the d4 and d8 position or (mutually exclusive with the d4 double bond) a hydroxylation at the C4 carbon atom. Another variable modification that can be detected is a hydroxylation at the C2 of the acyl chain. B, The depicted sphingolipid subclasses from pollen tubes grown under different temperature regimes were investigated. Abundances of the lipid subclasses were measured by UPLC-nanoESI-MS/MS and normalized to their respective values after 3 h RT. C, For each sphingolipid subclass, the percentage-wise occurrence of modifications was determined: HYD base C4, hydroxylation at C4 of the SPB; DB base d4, double bond at the Δ4 position of the SPB; DB base d8, double bond at the Δ8 position of the SPB; HYD FA C2, hydroxylation at C4 of the acyl chain; VLCFA, acyl chain derived from a very long chain fatty acid (C ≥ 20). *n* = 5. Error bars represent standard deviation. For statistical analysis, ANOVA was performed (significance level *α* = 0.05), followed by Post-hoc Tukey analysis. Results are presented as compact letter display of all pair-wise comparisons.

Sphingolipids are a highly diverse class of lipids. All sphingolipids consist of a sphingoid base (SPB) that can be subject to different modifications (Michaelson et al., 2016). If the SPB is linked to a 14–30 carbon acyl chain via an amide bond, a ceramide (Cer) is formed. Cers can be linked to a head group, consisting either of a hexose (giving rise to the subclass of hexosylceramides; HexCers) or a phosphate attached to an inositol. This inositol can then be further linked to glucuronic acid (GlcA) forming the core structure of glycosylinositolphosphoceramides, which can carry additional sugar residues. Both, the SPBs and the fatty acids can be further modified by double bonds or hydroxyl groups, SPBs and Cers can also be phosphorylated to either SPB-phosphates (SPBPs) or Cer-phosphates (Cer-Ps) (Figure 6A).

Overall, 96 sphingolipid species from 7 lipid subclasses were identified in the samples (Supplemental Datasets S4–S6). The abundance of sphingolipids in tobacco pollen tubes increased over time (Figure 6B) and HS led to an additional increase of all sphingolipid subclasses. Especially the levels of SPBP (5.8-fold increase when comparing HS 3 + 3 h to RT 6 h and 2.8-fold when comparing extended HS to HSR) and HexCer (30% and 31% increase, respectively) were elevated.

Sphingolipids of Arabidopsis pollen were previously described to be distinct from sporophytic tissues in having HexCer species that predominantly carry a double bond at the C4 position of the SPB instead of a hydroxy group (Luttgeharm et al., 2015). Also, in this study, after 3 h of growth, this double bond was detected in at least 90% of the HexCer species, in 74% of the SPB species and in 15% of the ceramide species (Figure 6C; for some lipid species, the position of the hydroxyl group could not unambiguously be determined). Interestingly, the occurrence of this modification was slightly reduced under HS. Instead, the relative abundance of species containing a hydroxyl group at this position increased (by 31% when compared with HS 3 + 3 h to RT 6 h). Another major change observable under HS was the increased hydroxylation at the C2 of the acyl chain of hexosyl-*N*-acetylhexosaminyl-GlcA-IPCs (Hex-HexNAc-GlcA-IPCs), which was detected in 45% of all Hex-HexNAc-GlcA-IPC species after 6 h growth at RT and was increased to 72% under HS.

### Levels and composition of free sterols and sterol conjugates are altered under HS conditions

Similar to sphingolipids, sterol lipids are important structural lipid constituents of membranes, especially the plasma membrane, and are important for membrane microdomain (lipid raft) formation. Through this, they are involved in diverse cellular processes, including polar cell growth (Simons and Ikonen, 1997; Beck et al., 2007; Simon-Plas et al., 2011; Mamode Cassim et al., 2019).

**Figure 7:**
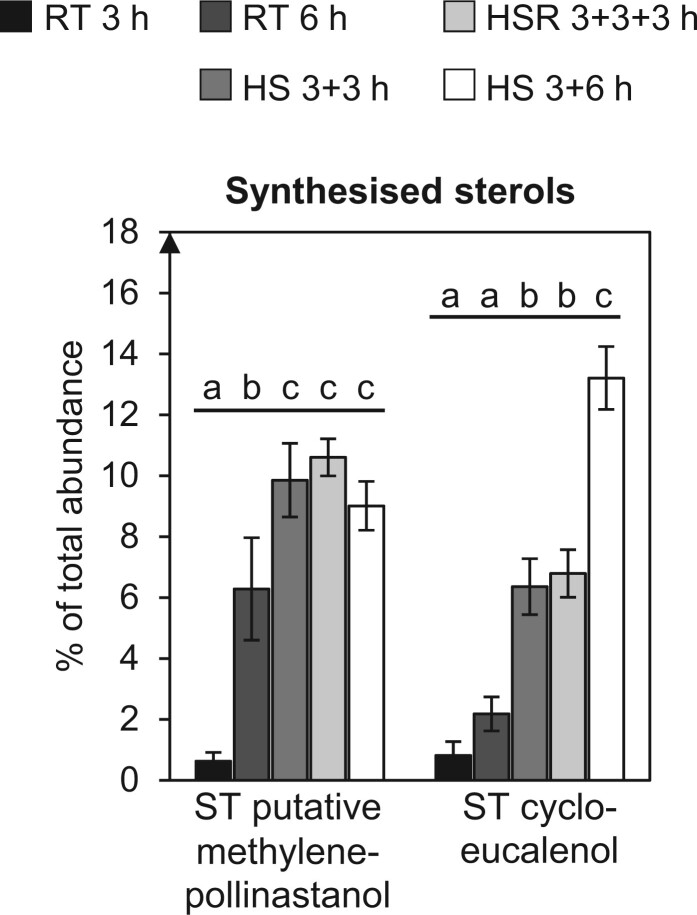
The relative amounts of newly synthesised free sterol species increase under HS. Only two sterol species are newly synthesised in growing tobacco pollen tubes, a sterol with an exact mass of 412,37 Da that is putatively methylenepollinastanol, and cycloeucalenol. Presented are relative abundances of the respective sterol based on the total ion current as measured by GC–MS, pollen tubes were grown under different temperature regimes. *n* = 5. Error bars represent standard deviation. For statistical analysis, ANOVA was performed (significance level *α* = 0.05), followed by post hoc Tukey’s analysis. Results are presented as compact letter display of all pair-wise comparisons in increasing order.

The profile of free sterols in pollen from various species differs greatly from that in leaves or other sporophytic tissues and it was shown that the sterol synthesis pathway in growing tobacco pollen tubes is truncated with only two species being de novo synthesised: presumably methylenepollinastanol and its precursor cycloeucalenol (Villette et al., 2015; Rotsch et al., 2017). Relative abundance of these sterol species increases during pollen tube growth (Rotsch et al., 2017), as can be seen in the presented data (Figure 7 and Supplemental Datasets S7 and S8). Especially cycloeucalenol accumulated strongly under HS and less at RT. After HSR, increased accumulation ceased and levels remained constant.

**Figure 8:**
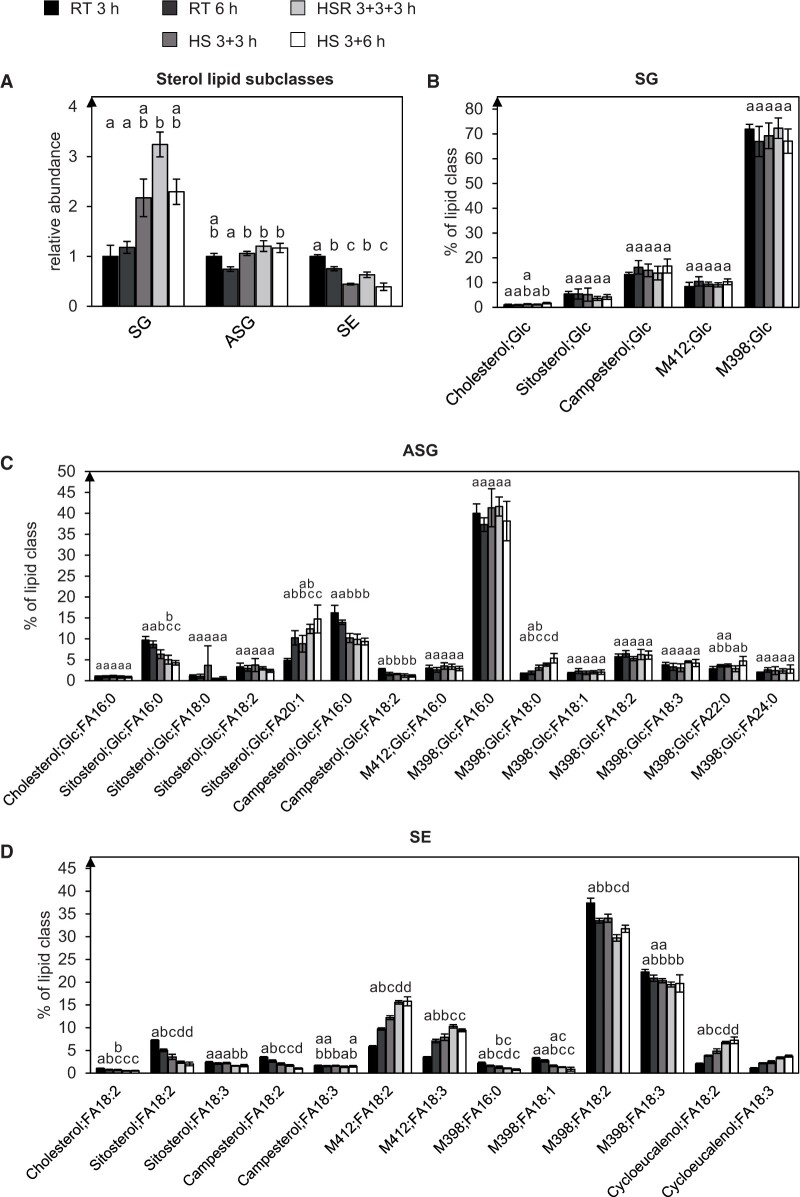
SGs accumulate under HS, while SEs decrease. A, The depicted sterol lipid subclasses from pollen tubes grown under different temperature regimes were investigated. Abundances of the lipid subclasses were measured by UPLC-nanoESI-MS/MS and normalized to their respective values after 3 h RT. B–D, Molecular species within the individual subclasses are given as mol % of all detected lipid species per subclass. Only major lipid species (>1% of total) are depicted. M398 could represent methylenecholesterol or Δ5,24-ergostadienoland, and M412 stigmasterol, the putative methylenepollinastanol, isofucosterol or 24-methylenelophenol. *n* = 5. Error bars represent standard deviation. For statistical analysis, ANOVA was performed (significance level *α* = 0.05), followed by post hoc Tukey’s analysis. Results are presented as compact letter display of all pair-wise comparisons in increasing order.

The relative levels of sterylglycoside (SG) and acylsterylglycoside (ASG) were also strongly increased by 84% and 42%, respectively, when comparing 3 h of HS to the control treatment. Sterol ester (SE) species on the other hand decreased in their relative levels (Figure 8 and Supplemental Datasets S10 and S11). When comparing prolonged heat treatment with HSR, HSR caused SE levels to increase again.

### Several central metabolites increase under HS

**Figure 9:**
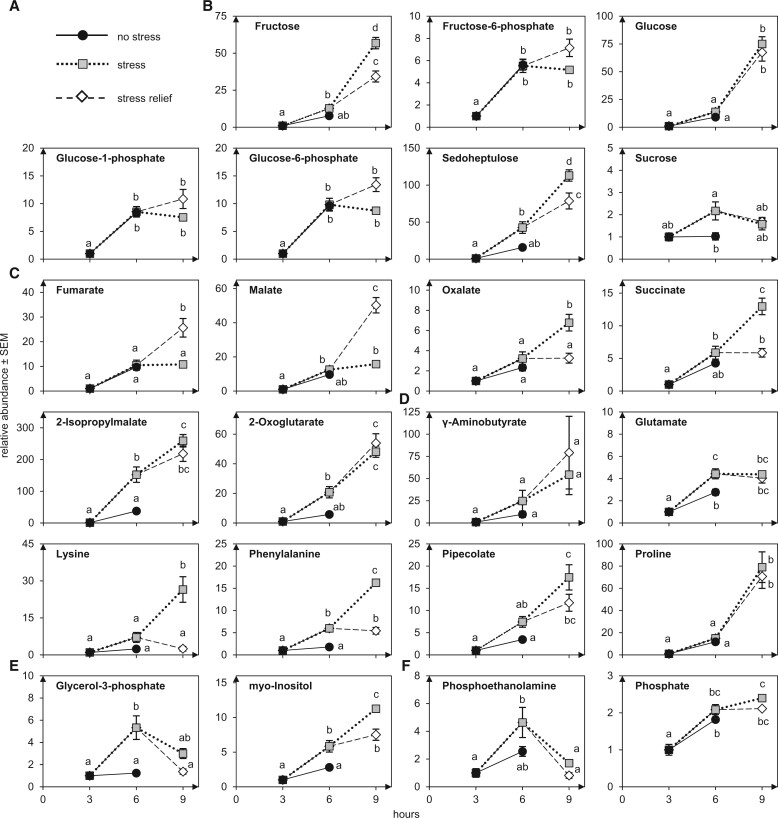
The abundance of several metabolites is affected by HS. A, Legend: black line and filled black circles: no stress (RT 3 h and RT 6 h); dotted line and gray squares: HS (3 + 3 h and 3 + 6 h); dashed lines and white rhombus:HSR (3 + 3 + 3 h). Shown are a selection of detected sugars (B), a selection of detected organic acids (C), a selection of detected amino acids (D), a selection of detected polyols (E) and others (F). *n* = 5. Metabolites are normalized to the value at 3 h. Error bars represent standard deviation. For statistical analysis, ANOVA was performed (significance level *α* = 0.05), followed by post hoc Tukey’s analysis. Results are presented as compact letter display of all pair-wise comparisons in increasing order.

In our study, we also extracted hydrophilic metabolites from pollen tubes grown under the five temperature regimes (Figure 9A). As pollen tubes cannot be easily washed, some liquid attached to the tubes that could contain secreted metabolites was included in the measurements. We identified 51 metabolites by GC–MS that are mostly part of central metabolism including organic acids, amino acids, and sugars (Supplemental Datasets S12 and S13). In addition, 17 so far unidentified markers were found. Values of all time points were normalized to the values after 3 h of RT. While most metabolites accumulated during prolonged pollen tube growth, not all of these were affected by HS. Sugar levels were only mildly altered with the exception of the seven-carbon sugar sedoheptulose that increased three-fold and sucrose that increased two-fold (Figure 9B). Among the organic acids, an increase in 2-isopropylmalate, an intermediate in leucine biosynthesis, and 2-oxoglutarate was observed (Figure 9C). Interestingly, fumarate and malate were not affected by HS but strongly increased after stress relief. Most amino acids increased during HS, including the noncodogenic amino acids β-lactate, γ-amino butyrate, and pipecolate (Figure 9D). Further metabolites that show an increase under HS include, for example glycerol-3-phosphate (Figure 9E) and PE (Figure 9F).

### HS leads to strong alteration on the transcriptome level

**Figure 10:**
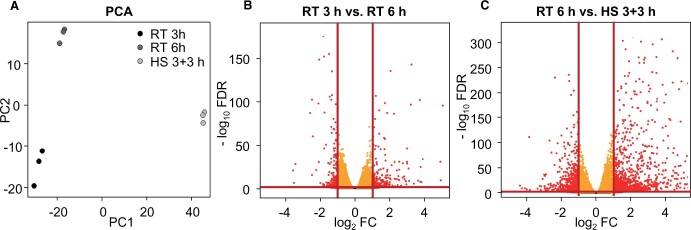
Analysis of transcriptome data. mRNA levels from pollen tubes grown under different temperature regimes were investigated by RNA sequencing. A, PCA of RT 3 h, RT 6 h and HS 3 + 3 h. B and C, Volcano plots of RT 3 h versus RT 6 h and RT 6 h versus HS 3 + 3 h, respectively. Red lines indicate threshold for differential expression (abs. log_2_ FC > 1, outside of vertical lines and FDR < 0.005, above horizontal line). *n* = 3.

To assess whether the different adaptations are reflected on a transcriptional level, transcriptome analyses of pollen tubes grown for 3 or 6 h at RT or 3 h at RT followed by 3 h at HS were performed by RNA sequencing (Supplemental Dataset S14). The corresponding principal component analysis (PCA) plot is shown in Figure 10A.

Overall, 26,743 genes were detected, 24,013 of which with more than 0.5 counts per million (CPM) in at least 2 libraries and 21,241 of these could be assigned a UniProt protein ID (Table 1). The respective protein sequences were then blasted against the TAIR 10 Arabidopsis protein library, and 19,798 tobacco proteins had Arabidopsis homologs with an Expect value (*E*-value) < 10^−5^. The respective Arabidopsis protein identifiers were later used for functional annotation.

**Table 1:** Number of detected and annotated genes in the respective analysis

Analysis	Detected genes	UniProt ID	Arabidopsis homolog
RT 3 h versus RT 6 h	23,043	20,459	19,162
RT 6 h versus HS 3 + 3 h	23,053	20,445	19,779
Total	24,013	21,241	19,798
Total unfiltered	26,743	–	–

Number of detected genes in total and in the two comparisons and number of total detected genes (unfiltered). For DE analyses, genes were filtered for a CPM < 0.5 in at least two of the respective analyzed libraries. Genes were annotated a UniProt identifier (UniProt ID), some genes could not be annotated, number of annotated genes is given. Annotated Tobacco proteins were blasted against Arabidopsis to find homologs, only hits with *E*-value < 10^−5^ were considered. Number of genes with an Arabidopsis protein homolog hit is given.

In the analysis of pollen tubes grown for 3 versus 6 h at RT, fold-changes (FCs) were calculated. About 561 genes were differentially expressed (defined as log_2_FC > 1; false discovery rate [FDR] ≤ 0.005), 306 of which were upregulated and 255 of which were downregulated (Figure 10B and Supplemental Dataset S15).

In the comparison of heat-stressed versus non-heat-stressed pollen tubes, 2,383 genes were differentially expressed, 1,570 of which were upregulated and 813 of which were downregulated (Figure 10C and Supplemental Dataset S16). For analyses of gene functions, we further explored the second comparison making use of the functional annotation of the Arabidopsis genome and proteome.

### GO-term analysis gives first insights

For gene ontology (GO) analysis, the GO terms assigned to the annotated Arabidopsis homologs were analyzed. For the analysis of RT 6 h versus HS 3 + 3 h, 20,370 genes were assigned with 5,994 different GO terms. Reads per kilo base per million mapped reads (RPKM) values of all genes belonging to the same GO term were summed up and averaged for the respective condition to calculate log_2_FCs.

A selection of the most changed GO terms between RT 6 h and HS 3 + 3 h is presented in Table 2, grouped by sub-ontology (for complete lists, see Supplemental Datasets S17–S20). Among them are several heat shock-related GO terms containing many upregulated genes and only few downregulated genes. The terms include “functions in Hsp90 protein binding,” “has unfolded protein binding,” and “involved in response to heat.” Another strongly changed GO term is “involved in response to ROS.” Other changed GO terms like “has pre-mRNA 3'-splice site binding” and “involved in positive regulation of mRNA splicing, via spliceosome” might hint at a role of alternative splicing under HS, which has been shown before, for example in tomato pollen (Keller et al., 2017). Interestingly, several changed GO terms suggest that auxin metabolism and/or signaling might be affected (“has auxin receptor activity,” “involved in auxin catabolic process,” and “involved in regulation of auxin biosynthetic process”).

**Table 2:** List of selected GO terms, their IDs, log_2_FCs (6 h 22°C versus 3 h 22°C + 3 h 37°C), *P*-values, the number of detected genes within the GO-term, and the number of DEGs (upregulated and downregulated) within the term

GO ID	GO term	log_2_FC	*P*-value	Genes	DEG	DEG
					up	down
Molecular function						
GO:0043621	Functions in protein self-association	4.09	1.4*E*−07	126	47	5
GO:0051879	Functions in Hsp90 protein binding	3.38	9.8*E*−08	21	12	0
GO:0051082	Has unfolded protein binding	2.20	5.7*E*−07	225	102	1
GO:0009916	Has alternative oxidase activity	1.44	7.1*E*−07	8	4	0
GO:0004765	Has shikimate kinase activity	1.06	0.0006	3	2	0
GO:0030628	Has pre-mRNA 3′-splice site binding	1.06	2.2*E*−05	20	7	0
GO:0000064	Has l-ornithine transmembrane transporter activity	−1.07	8.3*E*−05	5	0	2
GO:0005476	Has carnitine:acyl carnitine antiporter activity	−1.08	9.4*E*−05	3	0	2
GO:0008792	Has arginine decarboxylase activity	−1.35	9.2*E*−06	4	0	3
GO:0005244	Has voltage-gated ion channel activity	−1.46	0.0029	3	0	2
GO:0038198	Has auxin receptor activity	−2.01	0.0005	2	0	2
Cellular component						
GO:0089701	Located in U2AF	1.29	1.4*E*−05	18	7	0
Biological process						
GO:0009061	Involved in anaerobic respiration	9.36	2.6*E*−05	2	2	0
GO:0061077	Involved in chaperone-mediated protein folding	3.27	8.4*E*−07	33	11	2
GO:0010187	Involved in negative regulation of seed germination	2.73	5.1*E*−06	28	11	1
GO:0006457	Involved in protein folding	2.26	5.4*E*−07	202	97	1
GO:0048026	Involved in positive regulation of mRNA splicing, via spliceosome	1.80	1.0*E*−06	11	2	0
GO:0009852	Involved in auxin catabolic process	1.72	4.6*E*−05	2	1	0
GO:0000302	Involved in response to ROS	1.68	6.5*E*−08	76	46	0
GO:0034605	Involved in cellular response to heat	1.65	1.7*E*−06	47	20	0
GO:0009408	Involved in response to heat	1.29	6.6*E*−08	266	116	10
GO:0010600	Involved in regulation of auxin biosynthetic process	1.17	0.0032	4	3	0
GO:0010508	Involved in positive regulation of autophagy	1.11	7.8*E*−06	14	3	0
GO:0043618	Involved in regulation of transcription from RNA polymerase II promoter in response to stress	1.06	1.3*E*−05	22	9	0
GO:0033388	Involved in putrescine biosynthetic process from arginine	−1.33	9.6*E*−06	6	0	3
GO:0071456	Involved in cellular response to hypoxia	0.743	7.12*E*−07	211	67	11
GO:0009446	Involved in putrescine biosynthetic process	−1.33	9.6*E*−06	6	0	3
GO:0006527	Involved in arginine catabolic process	−1.33	9.8*E*−06	7	0	3

A selection of GO terms grouped by sub-ontologies (molecular function, cellular component, and biological process) that show a strong log_2_FC between pollen tubes grown for 6 h at 22°C and tubes grown for 3 h at 22°C followed by 3 h at 37°C. GO terms were quantified summing RPKM values of all detected genes within one GO term (number of genes is given) for the respective condition (6 h 22°C or 3 h 22°C + 3 h 37°C) and log_2_FC of the summed RPKMs was calculated. *P*-values were calculated using Student’s *t* test on summed RPKM values of the two conditions. Number of DEGs (log_2_FC > |1|, FDR < 0.005 according to DE analysis with EdgeR, Supplemental Datasets S12–S15) within the GO term are given.

### Transcripts of transcriptional regulators are strongly affected

The GO term analysis revealed the term “has transcription coactivator activity” to be 3.2-fold upregulated. The terms “functions in transcription regulatory region DNA binding” and “has DNA-binding transcription factor activity” showed 34 of 395 and 72 of 654 differentially upregulated, but also 31 and 15 downregulated genes, respectively. The total transcript abundance within these terms was, however, only little changed (Supplemental Dataset S19). To take a closer look at putative transcriptional regulators important for HS adaptation, transcript data were mined for genes with homology to Arabidopsis transcriptional regulators according to the Arabidopsis Plant Transcription Factor (TF) Database with 2,192 gene entries (Pérez-Rodríguez et al., 2010; Supplemental Dataset S21). Overall, 1,319 tobacco genes could be assigned to transcriptional regulators (117 upregulated and 42 differentially downregulated; Table 3 and Supplemental Dataset S22). The highest proportion of upregulated genes was found among the heat shock TFs (HSF, 9 out of 19) and DNA-binding protein phosphatases (DBP, 6 out of 10). Furthermore, the TF families, MYB-related and AP2-EREBP, showed a comparably high number of upregulated genes (14 and 9, respectively).

**Table 3:** List of all detected TF families with DEGs

TF family	Type	Arabidopsis genes	Detected genes	DEG up	DEG down
ABI3VP1	TF	57	27	1	1
Alfin-like	TF	7	10	0	1
AP2-EREBP	TF	146	34	9	1
ARF	TF	23	15	2	1
ARID	Other	10	17	1	1
AUX/IAA	Other	28	1	1	0
BES1	TF	8	10	0	1
bHLH	TF	136	44	5	3
bZIP	TF	70	49	1	5
C2C2-CO-like	TF	17	4	0	1
C2C2-GATA	TF	29	13	2	0
C2H2	TF	99	43	3	3
C3H	TF	68	90	7	1
CCAAT	TF	43	39	3	0
Coactivator p15	Other	3	2	2	0
CSD	TF	4	3	2	0
DBP	TF	4	10	6	0
EIL	TF	6	8	2	0
FAR1	TF	17	26	2	1
FHA	TF	17	20	3	0
G2-like	TF	40	20	2	0
GNAT	Other	32	34	1	0
GRAS	TF	33	17	3	3
HB	TF	91	19	1	0
HSF	TF	23	19	9	0
LOB	TF	43	14	1	0
LUG	Other	2	6	1	0
MADS	TF	105	30	2	2
MBF1	Other	3	7	2	0
mTERF	TF	34	11	1	1
MYB	TF	147	55	2	2
MYB-related	TF	65	71	14	1
NAC	TF	104	26	3	0
Orphans	TF	83	35	4	1
PHD	Other	45	92	3	1
Pseudo ARR-B	Other	5	5	1	1
RWP-RK	TF	14	10	2	1
SET	Other	33	46	3	0
Sigma70-like	TF	6	6	3	0
SNF2	Other	38	57	0	3
SWI/SNF-BAF60b	Other	16	10	1	0
TCP	TF	24	5	0	1
Tify	TF	15	9	0	1
TRAF	Other	25	16	0	2
TUB	TF	10	15	2	0
ULT	TF	2	4	0	1
VOZ	TF	2	4	1	0
WRKY	TF	72	22	3	1

List of all detected TF families that contain DEGs. As reference, the Arabidopsis Plant TF Database PlnTFDB with 2,192 gene entries was used (Pérez-Rodríguez et al., 2010; accessed on 4 June 2020). Given are the TF family names, their type (Other = other transcriptional regulator), the number of Arabidopsis genes belonging to the respective family, the number of detected Tobacco genes putatively belonging to the respective family and the number of DEGs (log_2_FC > |1|, FDR < 0.005) within the family.

### Transcripts related to lipid metabolism are barely affected by HS

To find out whether lipid adaptations are mirrored on a transcriptional level, a list of 746 Arabidopsis genes with a role or putative role in glycerolipid, glycerophospholipid, sphingolipid, and sterol lipid metabolism was compiled from several sources (see “Materials and methods” and Supplemental Dataset S23). Comparing our transcript data to this list, 713 tobacco transcripts were assigned a putative function in lipid metabolism (Supplemental Dataset S24). Of these, only 43 genes were differentially expressed, 20 of which were upregulated, and 23 downregulated (Table 4).

**Table 4:** List of DEGs with putative involvement in lipid metabolism

Tobacco gene	Tobacco protein	Arabidopsis gene	Arabidopsis protein	Putative function	log_2_FC	FDR
Acetyl CoA and fatty acid synthesis						
LOC107779989	A0A1S3YUM8	AT1G23800	ALDH2B7	Aldehyde dehydrogenase	1.59	1.8*E*−23
LOC107811394	A0A1S4BSN2	AT1G24360	KAR	Ketoacyl-ACP reductase	1.24	8.1*E*−08
LOC107789406	A0A1S3ZQ73	AT2G38040	α-CT/CAC3	Carboxyltransferase alpha subunit of heteromeric ACCase	−1.02	9.0*E*−22
LOC107829909	A0A1S4DHM5	AT5G10160	HAD	Hydroxyacyl-ACP dehydrase	−1.06	5.2*E*−08
LOC107789890	A0A1S3ZSC9	AT5G54960	PDC2	Pyruvate decarboxylase	−1.215	5.8*E*−04
LOC107769281	A0A1S3XVK6	AT1G65290	MTACP1	ACP, mitochondrial	−1.47	5.2*E*−06
LOC107790423	A0A1S3ZU33	AT4G13050	FATA2	Acyl-ACP thioesterase A	−1.17	0.0166
LOC107818291	A0A1S4CF02	AT2G34590	PDH (E1 beta)	Pyruvate dehydrogenase beta subunit	−1.57	1.1*E*−22
Fatty acid elongation, desaturation and export						
LOC107791219	A0A1S3ZWG6	AT5G10480	PAS2/HCD	Hydroxyacyl-CoA dehydratase	1.14	3.6*E*−11
LOC107789797	A0A1S3ZRX0	AT2G28630	KCS12	3-Ketoacyl-CoA synthase	−1.28	0.0040
Fatty acid degradation						
LOC107829745	A0A1S4DH57	AT5G42250		Zinc-binding alcohol dehydrogenase family protein	−1.04	1.7*E*−28
LOC107785996	A0A1S3ZEK9	AT1G06290	ACX3	Acyl-CoA oxidase	−1.17	2.8*E*−31
Glycerolipid metabolism						
LOC107761528	A0A1S3X5P8	AT4G26770	CDP-DGS	CDP-DAG synthase	−1.05	2.8*E*−23
LOC107759316	A0A1S3WYH5	AT5G10170	MIPS3	*myo*-inositol-3-phosphate synthase	−1.17	1.5*E*−20
LOC107800182	A0A1S4AQG0	AT3G17770		Dihydroxyacetone kinase	1.96	5.7*E*−08
LOC107827585	A0A1S4DAJ3	AT3G05510		Phospholipid/glycerol acyltransferase family protein	1.10	2.2*E*−11
LOC107773804	A0A1S3Y9M0	AT4G30340	AK7	Diacylglycerol kinase 7	−2.13	8.5*E*−05
TG synthesis						
LOC107830919	A0A1S4DLP1	AT2G19450	DGAT1/TAG1	Acyl-CoA:diacylglycerol *O*-acyltransferase	2.86	4.5*E*−25
LOC107789692	A0A1S3ZR81	AT2G19450	DGAT1/TAG1	Acyl-CoA:diacylglycerol *O*-acyltransferase	2.50	1.8*E*−07
LOC107775049	A0A1S3YDY1	AT2G19450	DGAT1/TAG1	Acyl-CoA:diacylglycerol *O*-acyltransferase	2.10	1.7*E*−08
LOC107779882	U5JD04	AT3G51520	DGAT2	Acyl-CoA:diacylglycerol *O*-acyltransferase	1.39	0.0002
LOC107811379	A0A1S4BSB8	AT1G48300	DGAT3	Acyl-CoA:diacylglycerol *O*-acyltransferase	1.12	4.3*E*−12
Lipid droplet-associated proteins						
LOC107774779	A0A1S3YCP8	AT4G10020	HSD5	Steroleosin	6.97	3.2*E*−51
LOC107821969	A0A1S4CS64	AT3G01570	OLE5	Oleosin family protein	5.87	4.3*E*−21
LOC107804268	A0A1S4B436	AT1G67360	LDAP1	Lipid droplet associated protein	1.14	3.8*E*−127
LOC107776898	A0A1S3YJQ0	AT1G10740	LDAH1	Lipid droplet associated hydrolase	1.10	6.4*E*−45
LOC107780677	A0A1S3YX57	AT3G18570	OLE8	Oleosin family protein	−1.22	3.3*E*−26
Acylglycerol degradation						
LOC107786500	A0A1S3ZGS3	AT5G04040	SDP1	Patatin-like phospholipase family protein	1.09	3.6*E*−72
LOC107779568	A0A1S3YTI2	AT2G39420	MAGL8	Monoacylglycerol lipase (MAGL)	−1.11	0.0001
LOC107815121	A0A1S4C4N0	AT3G62860	MAGL12	Monoacylglycerol lipase (MAGL)	−1.15	2.4*E*−59
LOC107774655	A0A1S3YC93	AT4G18550	DSEL	Cytosolic DAD1-like acylhydrolase, *sn*-1-specific lipase	−1.50	0.0007
Further lipases						
LOC107789128	A0A1S3ZPC3	AT2G03140		Alpha/beta-hydrolases superfamily protein	1.92	1.4*E*−123
LOC107827574	A0A1S4D9U6	AT1G31480	SGR2	PA-preferring phospholipase A1 (PA-PLA1) ?	1.19	8.5*E*−10
LOC107811391	A0A1S4BSF2	AT3G48090	EDS1	Alpha/beta-hydrolases superfamily protein	1.16	3.7*E*−10
LOC107786539	A0A1S3ZGY2	AT1G71250	GDSL-like	GDSL-like lipase/acylhydrolase superfamily protein	−1.09	6.8*E*−86
LOC107797353	A0A1S4AGE5	AT2G26560	PLP2	Phospholipase A 2A	−1.26	7.3*E*−14
LOC107779233	A0A1S3YSB9	AT1G29120		Hydrolase-like protein family	−1.27	1.6*E*−53
LOC107799608	A0A1S4ANI7	AT1G29120		Hydrolase-like protein family	−1.29	2.2*E*−32
Steroid biosynthesis						
LOC107795609	A0A1S4AAX4	AT2G29390	SMO2-2	Sterol 4-alpha-methyl-oxidase 2-2	3.03	9.1*E*−11
LOC107795958	A0A1S4AC88	AT3G19820	DWF1	Cell elongation protein/DWARF1/DIMINUTO (DIM)	−1.06	0.0003
Sphingolipid biosynthesis						
LOC107767458	A0A1S3XPT1	AT1G14290	SBH2	Sphingobase C4-hydroxylase	1.01	1.1*E*−65
LOC107826684	A0A1S4D6W9	AT4G36830	ELO4	ELO family protein	−1.03	5.2*E*−07
LOC107808708	A0A1S4BIQ7	AT2G46210	SLD2	Sphingobase-D8 desaturase	−1.05	1.1*E*−85

List of all differentially expressed tobacco genes with a putative involvement in lipid metabolism. Given are the Tobacco gene name, the annotated UniProt ID Tobacco protein name, the Arabidopsis homolog with the highest sequence similarity (gene name and protein name), and the assigned putative Tobacco protein function according to the Arabidopsis homology. Differential expression analysis was performed with EdgeR, log_2_FCs, and FDRs are given.

This result already indicates that lipid adaptations are not strongly reflected on a transcriptional level. To take a closer look at the differentially expressed genes (DEGs), lipid genes were grouped according to their pathways.

### Fatty acid synthesis is not transcriptionally upregulated

Pollen tube growth requires fatty acid synthesis in the plastids to cope with the increasing need for membrane lipids in growing pollen tubes (Ischebeck, 2016). Under HS, pollen tubes seem to need slightly more membrane lipids, as several minor lipid classes, as well as TG, levels accumulated more at high temperature (Figures 2A and 5B). However, neither of the pathways leading to acetyl-CoA synthesis via the pyruvate dehydrogenase nor pyruvate decarboxylase seems to be strongly affected (Supplemental Dataset S24). The same is true for fatty acid synthesis itself, as only one relevant gene, one of six putative ketoacyl-ACP reductases (KAR) was upregulated. It was previously shown that downregulation of the ketoacylsynthase KASII/FAB1, which is responsible for the elongation from C16 to C18 acyl chains, leads to strongly increased levels of C16 acyl chains in Arabidopsis (Pidkowich et al., 2007). Interestingly, none of the four isoforms was considerably downregulated in tobacco pollen tubes under HS, despite the increase of 16:0 acyl chains across lipid subclasses (Figure 3).

What is more, no differentially expressed fatty acid desaturases (FADs) were detected in our screen. FAD2 and FAD3, the desaturases that catalyze in PC the desaturation from 18:1 to 18:2 and from 18:2 to 18:3, respectively, are not downregulated but several isoforms were slightly upregulated.

### The transcriptome does not reflect adaptations in glycerolipid and glycerophospholipid profiles

The expression levels of enzymes involved in glycerolipid metabolism gave contrasting results. While some individual isoforms were differentially expressed (Table 4), only few clear trends could be observed. Two putative PA phosphatases homologous to Arabidopsis LPP1/PAP1 had very high abundance levels and increased by 25% and 55% during HS, respectively. Also two of three CGI58-type lysophosphatidic acid (LPA) acyltransferases that are involved in membrane and neutral lipid homeostasis (Ghosh et al., 2009; James et al., 2010) were slightly upregulated by 32% and 35%, respectively (Supplemental Dataset S19). Likewise, no clear trends for genes involved in galactoglycerolipid metabolism were observed.

Transcripts important for sulfolipid metabolism were not detected, indicating again that these lipids do not occur in tobacco pollen tubes.

### Transcripts hint at a dynamic turnover of TG upon HS

Taking a closer look at TG metabolism, we found that DG acyltransferase 1 (DGAT1), DGAT2, and DGAT3 homologs are strongly transcriptionally upregulated after 3 h of HS. However, a homolog of the major TG lipase SUGAR DEPENDENT 1 (SDP1) is also upregulated, so is another putative TG lipase. Upregulated genes also include some known LD-localized proteins; a putative 11-β-hydroxysteroid dehydrogenase (HSD) is very strongly upregulated (125-fold), which is interesting as previously no HSDs were found on LDs of tobacco pollen tubes on the protein level (Kretzschmar et al., 2018). Also, an oleosin, a lipid droplet-associated protein (LDAP) and a putative lipid droplet-associated hydrolase (LDAH) (Kretzschmar et al., 2020) show upregulation (Table 4); while another putative oleosin family protein is downregulated. Some low but significant changes can be observed in the scaffold protein plant UBX-domain-containing protein (PUX10) that plays a role in the degradation of LD proteins (Deruyffelaere et al., 2018; Kretzschmar et al., 2018). All four detected putative homologs show slight upregulation (Supplemental Dataset S24).

### Several genes involved in sterol and sphingolipid metabolism show differential expression

A transcript encoding a methylsterol monooxygenase 2 (SMO2)-like isoform showed eight-fold upregulation. SMO proteins are involved in sterol synthesis, to be more precise SMO2-1 and SMO2-2 are involved in the reaction from 24-methylenelophenol to episterol (precursor of campesterol, brassinosteroids, and brassicasterol) and from 24-ehtylenelophenol to δ7-avenasterol (that can be converted to isofucosterol, sitosterol, and stigmasterol). Furthermore, a putative δ(24)-sterol reductase shows two-fold upregulation.

Regarding sphingolipid metabolism, a Δ8-fatty-acid desaturase-like protein that shares 62% sequence identity with Arabidopsis SPHINGOID LONG CHAIN BASE DESATURASE 2 (SLD2) was two-fold downregulated, while other homologs of this enzyme showed upregulation (Table 4 and Supplemental Dataset S24). The transcript of a putative SPB hydroxylase 2 (SBH2) that inserts a hydroxyl group at the C4 position showed two-fold upregulation. One of three isoforms of dihydrosphingosine Δ-4 desaturases catalyzing a competing reaction, the insertion of a double bond at the Δ4 position, was downregulated by 31%, while the other isoforms displayed little change.

### DEGs suggest involvement of hormones in HS adaptation

Some other interesting DEGs that have not been covered so far are highlighted in Table 5. The gene displaying the strongest upregulation is annotated as “uncharacterized protein” in tobacco and its closest Arabidopsis homolog, AT3G10020, is annotated as “plant/protein.” Publications suggest that it is a stress-responsive gene. Strongest downregulation was observed for a homolog of BON ASSOCIATION PROTEIN 2 (BAP2), a reported inhibitor of programmed cell death (Yang et al., 2007).

**Table 5:** Selection of other DEGs of interest

Tobacco gene	Tobacco protein	Arabidopsis gene	Arabidopsis protein	Putative function	log_2_FC	log_2_CPM	FDR
LOC107766703	A0A1S3XM78	AT3G10020	Unknown protein	Plant/protein	12.07	3.38	0
LOC107778746	A0A1S3YRE7	AT5G05600	JAO2	2-Oxoglutarate (2OG) and Fe(II)-dependent oxygenase superfamily protein	10.23	3.19	0
LOC107817403	A0A1S4CCK8	AT3G22370	AOX1a	Alternative oxidase	9.05	0.41	9.24*E*−59
LOC107759427	A0A1S3WZ93	AT3G10020	Unknown protein	Plant/protein	7.89	2.57	2.52*E*−241
LOC107804414	A0A1S4B4D1	AT5G03370		Acylphosphatase family	6.96	−1.40	2.73*E*−15
LOC107774730	A0A1S3YCX4	AT3G09640	APX2	Ascorbate peroxidase	5.29	−1.31	3.20*E*−14
LOC107788327	A0A1S3ZM96	AT3G47520	MDH	Malate dehydrogenase	4.07	−0.51	5.52*E*−20
LOC107795525	A0A1S4AAK3	AT1G14130	DAO1	2-Oxoglutarate (2OG) and Fe(II)-dependent oxygenase superfamily protein	3.88	2.70	4.11*E*−184
LOC107801843	A0A1S4AVW8	AT5G47530		Auxin-responsive family protein	3.44	−0.79	2.59*E*−14
LOC107769851	A0A1S3XY09	AT3G21865	PEX22	Peroxin	3.40	0.28	4.42*E*−27
LOC107764611	A0A1S3XFN6	AT5G53120	SPDS3	Spermidine synthase	3.15	2.26	6.03*E*−95
LOC107808988	A0A1S4BJJ5	AT5G24240	ATPI4KGAMMA3	PI 4-kinase gamma-like protein	2.97	3.59	1.35*E*−219
LOC107817192	A0A1S4CB63	AT4G27510		2-Isopropylmalate synthase	2.36	−0.94	1.05*E*−07
LOC107762352	A0A1S3X8G8	AT1G49340	ATPI4KALPHA	PI 3- and 4-kinase family protein	2.36	1.92	7.99*E*−50
LOC107770164	A0A075EYT2	AT1G17710	PEPC1	Phosphoethanolamine/phosphocholine phosphatase	2.34	3.66	1.55*E*−173
LOC107808981	A0A1S4BJF1	AT1G17710	PEPC1	Phosphoethanolamine/phosphocholine phosphatase	2.00	5.00	9.42*E*−139
LOC107817132	A0A1S4CB76	AT1G17710	PEPC1	Phosphoethanolamine/phosphocholine phosphatase	1.50	2.74	3.69*E*−38
LOC107799822	A0A1S4APJ5	AT2G43020	PAO2	Polyamine oxidase	1.17	4.50	2.81*E*−72
LOC107768947	A0A1S3XUI8	AT1G65930	cICDH	Cytosolic NADP+-dependent isocitrate dehydrogenase	−1.01	3.04	6.39*E*−13
LOC107828000	A0A1S4DBY9	AT2G28350	ARF10	Auxin response factor	−1.46	0.28	1.39*E*−07
LOC107767522	A0A1S3XQ16	AT4G33430	BAK1	BRI1-associated receptor kinase	−1.47	−0.85	0.00091587
LOC107798522	T1WMC3	AT1G30135	JAZ8	Jasmonate-ZIM-domain protein	−1.49	−0.40	0.00010296
LOC107759371	A0A1S3WYR7	AT4G34710	ADC2	Arginine decarboxylase	−1.54	6.27	4.78*E*−241
LOC107819571	A0A1S4CJ33	AT1G02170	MC1	Metacaspase	−2.00	−1.31	0.00123282
LOC107832546	A0A1S4DR37	AT5G25260	FLOT2	Flotillin	−2.07	−0.44	1.32*E*−06
LOC107820452	A0A1S4CLU4	AT1G02170	MC1	Metacaspase	−2.57	1.00	7.54*E*−20
LOC107820447	A0A1S4CLX5	AT1G02170	MC1	Metacaspase	−2.72	−0.41	1.84*E*−09
LOC107805149	A0A1S4B6Y7	AT5G04200	MC9	Metacaspase	−2.86	−1.31	1.93*E*−06
LOC107824234	A0A1S4CZ94	AT2G19800	MIOX2	Myo-inositol oxygenase	−2.89	−0.54	2.29*E*−09
LOC107773293	A0A1S3Y7Q9	AT2G19800	MIOX2	Myo-inositol oxygenase	−3.17	0.20	1.86*E*−15
LOC107799833	A0A1S4APP9	AT2G45760	BAP2	BON association protein	−4.32	−0.46	7.69*E*−16

Selection of differentially expressed Tobacco genes (including the gene with the strongest upregulation and downregulation, respectively), the annotated UniProt ID Tobacco protein name, the Arabidopsis homolog with the highest sequence similarity (gene name and protein name), and the assigned putative Tobacco protein function according to the Arabidopsis homology. Differential expression analysis was performed with EdgeR, log2FC, log_2_CPM, and FDRs are given.

While auxins were already mentioned in the GO terms, some other hormones might also play a role in HS adaptation of tobacco pollen tubes. Strong upregulation was observed for a homolog of Arabidopsis JASMONATE-INDUCED OXYGENASE2 (JAO2), annotated as a protein with similarity to flavonol synthases and involved in the detoxification of polycyclic aromatic hydrocarbons (Hernández-Vega et al., 2017). JASMONATE-ZIM-DOMAIN PROTEIN 8 (JAZ8) on the other hand was transcriptionally downregulated. The transcript of a putative homolog of BRI1-ASSOCIATED RECEPTOR KINASE 1 (BAK1), a receptor-like kinase in brassinosteroid sensing, was downregulated under HS.

## Discussion

### The tobacco pollen tube transcriptome shows expected but also unique adaptations to HS

As shown in several previous studies on plants, HS leads to a rapid and strong remodeling of the transcriptome (Kotak et al., 2007; Mittal et al., 2012; Rahmati Ishka et al., 2018). One reaction conserved across prokaryotes and eukaryotes is the upregulation of heat shock proteins (HSPs) (Jacob et al., 2017). Accordingly, most transcripts strongly upregulated in response to HS encode HSPs in this study, indicating that the applied temperature regime imposes HS to the pollen tubes (Supplemental Dataset S16). This upregulation has also been found to be a key response under HS in developing pollen, prior to pollen tube growth (Fragkostefanakis et al., 2016; Keller et al., 2018).

However, we also found differences between HS responses in tobacco pollen tubes and vegetative tissues of Arabidopsis, as several homologs of heat-induced genes in Arabidopsis are expressed, but not upregulated upon HS in tobacco pollen tubes. These include genes encoding for LATE EMBRYOGENESIS ABUNDANT proteins, which protect other proteins and membranes especially during desiccation but are also upregulated by HS (Priya et al., 2019). On the contrary, we found genes upregulated that have so far not been associated with HS. These include, for example a flotillin-like gene homologous to Arabidopsis FLOT2 (Table 5). Flotillins are involved in formation of membrane microdomains (Haney and Long, 2010; Li et al., 2012). These data suggest that flotillins might also play an important role during pollen tube growth, maybe especially so under stress. The presence of sterol-rich membrane microdomains containing flotillin-like protein in rice pollen has recently been shown (Han et al., 2018).

### Heat-induced glycerolipid remodeling does not appear to be strongly transcriptionally controlled

The expression data shows no conclusive picture as to how the membrane lipid composition is altered. Nevertheless, several conclusions can be drawn from membrane lipid remodeling: Especially striking is the increase of the saturated acyl chains 16:0 and 18:0, while the ratio of monounsaturated to polyunsaturated acyl chains is not severely altered. This result suggests a regulation of fatty acid synthesis in the plastids, where it is determined if the acyl carrier protein (ACP)-connected acyl chains are initially elongated and desaturated. Only after elongation and desaturation from 16:0 to 18:1, the acyl chains can be precursors for further extraplastidial desaturation.

Arabidopsis and likely also other plant species harbor three types of KAS with only one, KASII/FAB1, being responsible for the elongation of 16:0-ACP to 18:0-ACP (Wu et al., 1994; Pidkowich et al., 2007). KASII/FAB1 would thus pose a good target for regulation. Regulation of the Δ9 desaturase FAB2 could be a further factor, as it introduces the first double bond to form 18:1. Furthermore, thioesterases might play a role, as knockout of FATB leads to a strong reduction of saturated acyl chains (Bonaventure et al., 2003) and knockdown of the two FATA genes also influences acyl composition (Moreno-Pérez et al., 2012).

One can speculate that during HS the de novo synthesised fatty acids are even stronger saturated than reflected by the membrane lipid composition, as some acyl chains were likely already synthesised prior to HS. This would imply a rapid and strong adaptation of the above-mentioned enzymes, likely through protein degradation, posttranslational modification, or a direct influence of temperature on the enzymatic activities. A transcriptional regulation is, however, unlikely, as respective transcripts were little or not changed (Supplemental Dataset S24).

### Role of PS and galactoglycerolipids in HS adaptation

Some lipid subclasses increased comparably stronger under HS. This is especially true for PS and DGDG. PS is found in the cytosolic leaflets of the plasma membrane and in endosomes and is negatively charged. It can form nanodomains in the plasma membrane that are important for Rho signaling (Platre et al., 2019). These small G proteins have also been shown to be important for the regulation of pollen tube growth (Scholz et al., 2020), but it remains to be studied if they also depend on PS, how PS is distributed in the pollen tube, and if this distribution changes under HS.

Galactoglycerolipids are the most abundant lipids of thylakoids and there is strong galactoglycerolipid remodeling under temperature stress in Arabidopsis (Chen et al., 2006; Higashi et al., 2015) and tomato leaves (Spicher et al., 2016). In agreement with studies concerning other classes of membranes lipids, the level of unsaturation in galactoglycerolipids is inversely associated with growth temperature. Furthermore, heat acclimation at 38°C in wild-type Arabidopsis increases DGDG, the DGDG-to-MGDG ratio, and the saturation level of DGDG (Higashi et al., 2015). Also, a role for galactoglycerolipids in acquired thermotolerance was already described in a study in 2006, when the authors did a mutant screen for plants defective in the acquisition of thermotolerance and found a mutant of *DGD1* (Chen et al., 2006). While pollen tubes contain plastids, these harbor no thylakoids (Staff et al., 1989) and galactoglycerolipids in pollen tubes were discussed to be especially important in extraplastidial membranes, where they are also formed in phosphate-starved vegetative tissues (Härtel et al., 2000). Evidence for the abundance of galactoglycerolipids in the male gametophyte comes from glycerolipid profiling of lily (*Lilium longiflorum*) pollen tubes before and after elongation revealing a 5.7-fold increase in DGDG and a 2.8-fold increase in the MGDG levels (Nakamura et al., 2009). The use of the MGDG synthase inhibitor galvestine-1 further highlighted an important role of galactoglycerolipids in pollen tube growth and the use of a specific antibody indicated a DGDG localization at the periphery of Arabidopsis pollen tubes most probably at the plasma membrane (Botté et al., 2011). In addition, the fact that galactoglycerolipids account for 11% of all membrane-forming glycerolipids in tobacco pollen tubes (Müller and Ischebeck, 2018) speaks for their presence in extraplastidial membranes.

### Transcriptome and lipidome suggest dynamic adaptations in LD-turnover and TG metabolism

While transcripts involved in lipid metabolism were mostly unaffected by HS, transcripts coding for genes involved in TG turnover and LD biology showed more dynamic adaptations to HS (Supplemental Dataset S24). An increase of TG levels under HS has already been observed, for example, in the algal species *Nannochloropsis oculata* (Converti et al., 2009), *Ettlia oleoabundans* (Yang et al., 2013), *Coccomyxa subellipsoidea* C169 (Allen et al., 2018), or *Chlamydomonas reinhardtii* (Légeret et al., 2016), but also in Arabidopsis leaves (Higashi et al., 2015; Shiva et al., 2020) and seedlings (Mueller et al., 2015), and tomato fruits (Almeida et al., 2021). In Arabidopsis seedlings, PHOSPHOLIPID:DIACYLGLYCEROL ACYLTRANSFERASE1 (PDAT1), an enzyme transferring a fatty acid from PC to DG to yield TG, is necessary for heat-induced TG accumulation. Also, the *pdat1* mutant seedlings were more sensitive to HS, indicating that PDAT1-mediated TG accumulation mediates thermotolerance (Mueller et al., 2017). Another interesting fact hinting at an involvement of not just TG but LDs as organelles in HS adaptations is the 125-fold upregulation of a putative HSD5 homolog (Table 4) suggesting an involvement of LDs in HS adaptation beyond TG accumulation. HSDs, also called steroleosins, are important for plant development and are involved in stress responses as well as wax metabolism (Li et al., 2007; Zhang et al., 2016; Shao et al., 2019). Steroleosins are presumably involved in brassinosteroid metabolism (Li et al., 2007) but were previously not found on LDs of tobacco pollen tubes (Kretzschmar et al., 2018). In this study, only two transcripts with very low expression levels were detected in nonstressed conditions.

### Changes in sterol lipid and sphingolipid metabolism might contribute to heat adaption

In contrast to TG that increases during HS and might therefore act as a sink for acyl chains, SE species are decreased, indicating that free sterols are released during heat adaptation and could help to adapt membrane fluidity (Dufourc, 2008). Free sterols could furthermore be converted to SG and ASG species, which were shown to increase upon HS. So far, no plant sterol esterase has been described (Ischebeck et al*.*, 2020) and it is not clear if SE breakdown is regulated transcriptionally.

Alterations in sphingolipid composition on the other hand could at least in part be explained by changes in transcript levels—the slight increase of hydroxylation versus desaturation at the C4 position of the SPB in HexCers is in line with the respective changes in the transcripts coding for the responsible enzymes (Table 4 and Supplemental Dataset S24). The increased hydroxylation of the SPB moiety in HexCers and the acyl chain in Hex-HexNAc-GlcA-IPCs could stabilize sphingolipid-containing microdomains through their ability to form lateral hydrogen bonds with other sphingolipids or free sterols (Mamode Cassim et al., 2019).

The strong increase of SPBP on the other hand is not reflected on the transcript level as none of the most abundant SPB kinases are strongly upregulated (Supplemental Dataset S24). The production of SPBP is thought to play a role in balancing the levels of putatively toxic SPBs. Furthermore, SPB breakdown requires its phosphorylation to SPBP followed by degradation to PE and palmitic aldehyde by SPBP lyase. Furthermore, SPBP could have signaling function (Puli et al., 2016).

### Metabolomic adaptations might facilitate thermotolerance

In previous studies on heat-stressed shoot tissues of Arabidopsis (Kaplan et al., 2004; Harsh et al., 2016; Zinta et al., 2018; Lawas et al., 2019), it was found that different sugars accumulated, including glucose, fructose, and in some cases sucrose. In tobacco pollen tubes, the heat-induced accumulation of sugars was rather modest in comparison and the somewhat higher increase in sucrose has to be considered with care, as sucrose was also contained in the growth medium (Figure 9). Interestingly though, we found a strong increase in sedoheptulose, a seven carbon sugar normally occurring in its phosphorylated form in the Calvin–Benson cycle and the pentose phosphate pathway. It is unclear if this sugar has any protective function, but it was also found to highly accumulate in the alga *Phaeodactylum tricornutum* under nitrogen deprivation (Popko et al., 2016).

In addition, an increase of several free amino acids was observed under HS (Figure 9). Proline levels, however, stayed relatively stable and although it accumulates very strongly after 6 h of pollen tube growth, it does so under normal as well as stress conditions. This is interesting, as proline was found to be clearly heat-inducible in studies on shoot tissues (Harsh et al., 2016; Zinta et al., 2018; Lawas et al., 2019). However, a study on potato (*Solanum tuberosum*) leaves showed that HS alone does not have substantial influence on proline levels, neither in stress-susceptible nor in tolerant potato cultivars. In the study, proline only accumulated after drought or combined HS and drought stress (Demirel et al., 2020). The same study observed an increase in lysine in one of the stress-susceptible cultivars following HS. It is possible that pipecolate, similar in structure to proline, plays a role in adaptation to heat in pollen tubes, as pipecolate was more heat-responsive than proline in this study. Pipecolate was also shown to strongly accumulate during tobacco pollen development (Rotsch et al., 2017) and it increases in shoot tissues upon pathogen infection (Návarová et al., 2013; Ding et al., 2016).

## Conclusion

Adaptation of tobacco pollen tubes to HS appears to be a complex and multi-layered trait that requires intricate interplay of different cellular processes. A rapid lipid remodeling that is not controlled transcriptionally is equally as important as metabolomic adaptations and transcriptional changes. Therefore, this study with several large datasets is a useful resource for future studies on HS tolerance.

## Materials and methods

### Plant material and growth conditions

Tobacco (*N.* *tabacum* L. cv. Samsun-NN) plants were grown in the greenhouse as previously described (Rotsch et al., 2017): plants were kept under 14 h of light from mercury-vapor lamps in addition to sunlight with light intensities of 150–300 µmol m^−2^ s^−1^ at flowers and 50–100 µmol m^−2^ s^−1^ at mid-height leaves. Temperature was set to 16°C at night and 21°C during the day with a relative humidity of 57%–68%.

Pollen was first pooled for each experiment and was derived from 5 to 20 plants and 50 to 200 flowers. The pooled pollen was weighed and rehydrated for 10 min in liquid pollen tube medium (5% [w/v] sucrose, 12.5% [w/v] PEG-4000, 15 mM MES-KOH pH 5.9, 1 mM CaCl_2_,1 mM KCl, 0.8 mM MgSO_4_, 0.01% [w/v] H_3_BO_3_, 30 µM CuSO_4_) modified from (Read et al., 1993). Aliquots of the suspension containing a defined amount of pollen (indicated below for each experiment) were spread onto cellophane foil (Max Bringmann KG, Germany) and placed on 50 mL of solid pollen tube medium (2% [w/v] Agarose, 5% [w/v] sucrose, 6% [w/v] PEG-4000, 15 mM MES-KOH pH 5.9, 1 mM CaCl_2_,1 mM KCl, 0.8 mM MgSO_4_, 0.01% [w/v] H_3_BO_3_, 30 µM CuSO_4_) inside square petri dishes (120 × 12017 mm with vents, Greiner Bio-One, Kremsmünster, Austria), sealed with MicroporeTM (3 M; Saint Paul, MN, USA). All pollen tubes were grown for 3 h at RT. Control pollen tubes were kept at RT for another 3 h, while heat stressed-pollen tubes were transferred to 37°C for 3 h. For HSR, pollen tubes were then transferred to RT again for the next 3 h, while nonrelieved pollen tubes remained at 37°C. Pollen tubes were harvested using a spatula and forceps, and directly transferred into extraction solution for lipid or metabolite analysis. For RNA extraction, the material was immediately flash-frozen for further processing.

### Lipidomic analysis

For lipidomic analyses with an UPLC system coupled with a chip-based nanoESI source and a triple quadrupole analyzer for MS/MS (Herrfurth et al., 2021), 20 mg of freeze-dried pollen tube tissue were ground and extracted with 60:26:14 (v/v/v) propan-2-ol/hexane/water according to Markham et al. (2006). After extraction, the sample was dissolved in 0.8 mL 4:4:1 (v/v/v) tetrahydrofuran/methanol/water. UPLC-nanoESI-MS/MS analysis of molecular species from 36 different lipid subclasses was performed with lipid subclass-specific parameters as shown in Supplemental Table S1. For reverse-phase LC separation, an ACQUITY UPLC I-class system (Waters Corp., Milford, MA, USA) equipped with an ACQUITY UPLC HSS T3 column (100 × 1 mm, 1 μm; Waters Corp., Milford, MA, USA) was used. Solvent A was 3:7 (v/v) methanol/20 mM ammonium acetate containing 0.1% (v/v) acetic acid and solvent B was 6:3:1 (v/v/v) tetrahydrofuran/methanol/20 mM ammonium acetate containing 0.1% (v/v) acetic acid. All lipid subclasses were separated with binary 16 min gradients with a flow rate of 0.1 mL/min (except for TG where 0.13 mL/min were used) following the same scheme: respective start condition (Supplemental Table S1) held for 2 min, linear increase to 100% solvent B for 8 min, 100% solvent B held for 2 min and re-equilibration to start conditions in 4 min. Chip-based nanoESI was achieved with a TriVersa Nanomate (Advion, Ithaca, NY, USA) in the positive or negative ion mode, respectively (Supplemental Table S1). Lipid molecular species were detected with a 6500 QTRAP tandem mass spectrometer (AB Sciex, Framingham, MA, USA) by multiple reaction monitoring (MRM) with lipid subclass-specific parameters (Supplemental Table S1) and a dwell time of 5 ms. For glycerolipid and glycerophospholipid analysis, all mass transitions for the distinct lipid subclasses were measured for the putative lipid species having 16:0, 16:1, 16:2, 16:3, 17:0, 17:1, 17:2, 17:3, 18:0, 18:1, 18:2, 18:3, 19:0, 19:1, 19:2, 19:3, 20:0, 20:1, 20:2, 22:0, 22:1, 24:0, 24:1, 26:0, and 26:1 as acyl residues. For sphingolipid analysis, all mass transitions for the distinct lipid subclasses were measured for the putative lipid species having 18:0;O2, 18:1;O2, 18:2;O2, 18:0;O3, and 18:1;O3 as SPB residues and chain lengths from C16 to C28 as fatty acid residues that are saturated or monounsaturated and unhydroxylated or monohydroxylated. For sterol lipid analysis, all mass transitions for the distinct classes were measured for the putative lipid species having cholesterol, sitosterol, campesterol, stigmasterol/putative methylenepollinastanol/isofucosterol/24-methylenelophenol, 24-methylenecholesterol/Δ5,24-ergostadienol, and cycloeucalenol as steryl residue and the fatty acids as acyl residue as described above for the glycerolipid and glycerophospholipid analysis. To improve the chromatographic separation and mass spectrometric detection of distinct lipid subclasses, phosphate groups and hydroxyl groups of the respective lipid compounds were chemically modified by methylation (LPA, PA, PIP, and PIP_2_) modified from Lee et al. (2013) or by acetylation (Cer-P and SPBP) modified from Berdyshev et al. (2005), respectively. The calculation of the relative abundance of all detected lipid subclasses and of relative lipid subclass profiles is described in Supplemental Datasets S1–S6 and S9–S11. All calculations were made according to the following procedure.

1. Correction of the absolute peak area with the naturally occurring proportion of the ^13^C isotopes (isotopic correction factor, icf);
2. Addition of icf-corrected absolute peak area from corresponding mass transitions;
3. Total peak area of a lipid subclass (addition of all detected molecular species of the respective lipid subclass) and normalization to the values after 3 h of RT growth (relative abundance compared with RT 3 h); relative proportion of the lipid species-specific peak area on the total peak area of the respective lipid subclass (relative lipid subclass profile).

### Lipid extraction and fatty acid methyl esterification

For analyses by GC-FID, total lipids from pollen tubes grown from 20 mg of dry pollen were extracted by methyl-*tert*-butyl ether (MTBE) extraction (modified from Matyash et al., 2008). Pollen tubes were harvested into 2 mL of preheated propan-2-ol (75°C) and kept at 75°C for 5–10 min. About 0.05 mg triheptadecanoate (Merck KGaA, Darmstadt, Germany) in 50 µL chloroform as the internal standards for absolute quantification was directly added to the propan-2-ol. Propan-2-ol was transferred to a new vial and evaporated under N_2_ stream and the pollen tubes were covered with 2 mL of 3:1 (v/v) MTBE/methanol. Pollen tube tissue was disrupted with a spatula, vortexed, and then shaken at 4°C for 1 h. After 5 min of centrifugation at 1,000 × *g*, the supernatant was transferred to the vial with the evaporated propan-2-ol and 1 mL of 0.9% (w/v) NaOH was added. Samples were vortexed and centrifuged again before the upper phase was transferred to a new vial. Solvent was evaporated under a N_2_ stream and samples were dissolved in 250 µL of 65:56:8 (v/v/v) chloroform/methanol/water.

The total lipids were then separated by thin layer chromatography (TLC). About 80 µL of the dissolved samples were spotted with a TLC spotter to TLC plates (TLC Silica gel 60, Merck KGaA). For extraction of TG, plates were run in 80:20:1 (v/v/v) hexane/diethylether/acetic acid. The bands comigrating with the TG standard were scratched out. To obtain fatty acid methyl esters (FAMEs), samples were then subjected to 1 mL FAME reagent (2.5% [v/v] H_2_SO_4_, 2% [v/v] dimethoxypropane in 2:1 [v/v] methanol/toluene) and under constant shaking incubated at 80°C in a water bath for 1 h. The reaction was stopped by adding 1 mL of saturated NaCl solution and vortexing. FAMEs were then extracted by adding 1 mL of hexane, centrifuging 10 min at 2,000 × g, and transferring the upper phase to a new vial. Hexane was evaporated and samples were resuspended in 25 µL of acetonitrile for subsequent GC-FID analysis.

### Central metabolite and sterol extraction and derivatization

Primary metabolite and sterol extraction were performed as previously described (Rotsch et al., 2017). Per time point, pollen tubes grown from 5 mg of pollen were harvested and freeze-dried overnight. After tissue disruption, 500 µL extraction solution (32.25:12.5:6.25 [v/v/v] methanol/chloroform/water) was added per sample, vortexed, and incubated for 30 min at 4°C under constant shaking. Supernatant was transferred to a new tube and pollen tubes were extracted again with another 500 µL of extraction solution. Incubation was repeated and supernatants were combined. About 0.0125 mg *allo*-inositol in 0.5 mL water was added, incubated again at 4°C for 30 min, centrifuged 5 min at full speed, and the aqueous phase containing the metabolites was transferred. About 20 µL was evaporated under a N_2_ stream and used for derivatization with 15 µL of methoxyamine hydrochloride in pyridine (30 mg/mL) overnight at RT. Derivatization with 30 µL *N*-methyl-*N*-(trimethylsilyl) trifluoroacetamide (MSTFA) followed for at least 1 h to obtain methoxyimino and trimethylsilyl derivatives of the metabolites (Bellaire et al., 2014).

For the analysis of sterols, 2 mL of 3:1 v/v MTBE/methanol and 1 mL of 0.9% (w/v) NaCl were added to 200 µL of the organic phase and evaporated under a N_2_ stream. Samples were dissolved in 20 µL pyridine, 10 µL of which were used for MSTFA derivatization with 10 µL of MSTFA 1–6 h prior to analysis.

### GC-FID and GC–MS

GC-FID analysis of FAMEs was performed as described (Hornung et al., 2002): an Agilent GC 6890 system (Agilent, Waldbronn, Germany) coupled with an FID detector equipped with a capillary DB-23 column (30 m × 0.25 mm, 0.25 µm coating thickness, Agilent) was used. Helium served as the carrier gas (1 mL/min), with an injector temperature of 220°C. The temperature gradient was 150°C for 1 min, 150°C–200°C at 15 K min^−1^, 200°C–250°C at 2 K min^−1^, and 250°C for 10 min.

For quantification, peak integrals were determined using Agilent ChemStation for LC 3D systems (Rev. B.04.03) and used to calculate absolute amounts of TG as well as relative fatty acid contributions.

For the measurement of central metabolites and sterols, GC–MS measurements were performed as previously described in Touraine et al. (2019) for metabolites and Berghoff et al. (2021) for sterols. If the metabolites were not identified by an external standard, the spectra were identified with the Golm metabolome database (GMD) and the National Institute of Standards and Technology spectral library 2.0f. The chemical information on metabolites identified with the GMD can be obtained at http://gmd.mpimp-golm.mpg.de/search.aspx (Kopka et al., 2005). Due to the high levels, sucrose was measured in separate runs using only one-tenth of the sample as used for the regular runs.

Data were analyzed using MSD ChemStation (F.01.03.2357). Masses used for quantification are depicted in Supplemental Dataset S7.

### RNA extraction, library preparation, and sequencing

Total RNA was extracted from 200 mg of pollen tubes per sample using Spectrum Plant Total RNA Kit (Sigma-Aldrich, St. Louis, MO, USA). RNA-seq libraries were performed using the non-stranded mRNA Kit from Illumina (San Diego, CA, USA; Cat. No. RS-122-2001). Quality and integrity of RNA were assessed with the Fragment Analyzer from Advanced Analytical (Heidelberg, Germany) by using the standard sensitivity RNA Analysis Kit (Agilent, Santa Clara, CA, USA; DNF-471). All samples selected for sequencing exhibited an RNA integrity number >8. After library generation, for accurate quantitation of cDNA libraries, the fluorometric-based system QuantiFluordsDNA (Promega, Madison, WI, USA) was used. The size of final cDNA libraries was determined using the dsDNA 905 Reagent Kit (Fragment Analyzer, Advanced Bioanalytical) exhibiting a sizing of 300 bp on average. Libraries were pooled and sequenced on an Illumina HiSeq 4000 (Illumina), generating 50 bp single-end reads (30–40 Mio reads/sample). The data can be found under the GEO ID: GSE153474 and the link https://www.ncbi.nlm.nih.gov/geo/query/acc.cgi?acc=GSE153474.

### Raw read and quality check

Sequence images were transformed with Illumina software BaseCaller to BCL files, which was demultiplexed to fastq files using bcl2fastq 2.17.1.14. The sequencing quality was assessed using FastQC (version 0.11.5; https://www.bioinformatics.babraham.ac.uk/projects/fastqc/).

### Mapping and normalization

Samples were aligned to the reference genome *N.* *tabacum* (Ntab version TN90, https://www.ncbi.nlm.nih.gov/assembly/GCF_000715135.1/) using the STAR aligner (version 2.5.2a) (Dobin et al., 2013) allowing for two mismatches within 50 bases. Subsequently, reads were quantified for all Ntab version TN90 genes in each sample using featureCounts (version 1.5.0-p1) (Liao et al., 2014).

### Differential gene expression analysis

Read counts were analyzed in the R/Bioconductor environment (Release 3.10, www.bioconductor.org) using edgeR package (version 3.28.1) (Robinson et al., 2009; McCarthy et al., 2012) gene names were translated to UniProt protein identifiers. For more detailed functional analyses, these tobacco proteins were blasted against the TAIR 10 Arabidopsis protein library (TAIR10, https://www.arabidopsis.org/download_files/Proteins/TAIR10_protein_lists/TAIR10_pep_20101214) using Protein–Protein BLAST 2.5.0+ with a maximum target sequence of 1. Only those hits with an Expect value (*E*-value describes the amount of hits to be expected by chance for the respective database size) <10^−5^ were considered. The obtained Arabidopsis AGI-codes were then assigned GO-terms for GO-term analysis. The GO term annotations were obtained from the Arabidopsis Information Resource (www.arabidopsis.org) in a version updated on 1 January 2020.

To analyze genes with a putative involvement in lipid metabolism, a compiled list of lipid genes was generated using The Arabidopsis Acyl-Lipid Metabolism Website (Li-Beisson et al., 2013), KEGG pathway (https://www.genome.jp/kegg/pathway.html, latest update 10 March 2020), and genes from Kretzschmar et al. (2020), Kelly and Feussner (2016), and Luttgeharm et al. (2016).

### Statistical analyses

Statistical analyses were performed as indicated for the respective experiments. For multiple comparisons, analysis of variance (ANOVA) was performed, followed by post hoc Tukey analysis. Results are presented as compact letter display of all pair-wise comparisons. Unpaired two-sample *t* tests were performed if just two means were compared. Results are presented as * (*P* < 0.05), **(*P* < 0.01), and ***(*P* < 0.005).

## Accession numbers

The annotation file of all Nicotine Tobacco genes can be found within the file “GSE153474_GCF_000715135.1_Ntab-TN90_genomic_edited.gtf.gz” under the link to the GEO dataset (https://www.ncbi.nlm.nih.gov/geo/query/acc.cgi?acc=GSE153474).

## Supplemental data

The following materials are available in the online version of this article.

**Supplemental Table S1.** Parameters for lipid analysis by UPLC-nanoESI-MS/MS.

**Supplemental Dataset S1.** Glycerolipids: absolute peak areas.

**Supplemental Dataset S2.** Glycerolipids: icf-corrected peak areas (partially summed) on the molecular species level and the lipid subclass level.

**Supplemental Dataset S3.** Glycerolipids: relative peak areas as % of all icf-corrected peak areas of the respective lipid subclass.

**Supplemental Dataset S4.** Sphingolipids: absolute peak areas.

**Supplemental Dataset S5.** Sphingolipids: icf-corrected peak areas (partially summed) on the molecular species level and the lipid subclass level.

**Supplemental Dataset S6.** Sphingolipids: relative peak areas as % of all icf-corrected peak areas of the respective lipid subclass.

**Supplemental Dataset S7.** Free sterols: raw data.

**Supplemental Dataset S8.** Free sterols: relative levels.

**Supplemental Dataset S9.** Sterol conjugates: absolute peak areas.

**Supplemental Dataset S10.** Sterol conjugates: icf-corrected peak areas on the molecular species level and the lipid subclass level.

**Supplemental Dataset S11.** Sterol conjugates: relative peak areas as % of all icf-corrected peak areas of the respective lipid subclass.

**Supplemental Dataset S12.** Metabolite analysis: list of analytes.

**Supplemental Dataset S13.** Metabolite analysis: list of metabolites.

**Supplemental Dataset S14.** Transcriptomics: raw counts.

**Supplemental Dataset S15.** Transcriptomics: differential gene expression analysis 3 h 23°C versus 6 h 23°C.

**Supplemental Dataset S16.** Transcriptomics: differential gene expression analysis 6 h 23°C versus 3 h 23°C + 3 h 37°C.

**Supplemental Dataset S17.** Transcriptomics: GO term analysis.

**Supplemental Dataset S18.** Transcriptomics: GO term analysis—biological process.

**Supplemental Dataset S19.** Transcriptomics: GO term analysis—molecular function.

**Supplemental Dataset S20.** Transcriptomics: GO term analysis—cellular component.

**Supplemental Dataset S21.** List of Arabidopsis transcription regulators.

**Supplemental Dataset S22.** Transcriptomics: list of all detected putative transcription regulators.

**Supplemental Dataset S23.** List of Arabidopsis genes with a putative involvement in lipid metabolism.

**Supplemental Dataset S24.** Transcriptomics: list of all detected genes with putative lipid function.

## Supplementary Material

kiac127_Supplementary_Data

## Notes

H.E.K., A.H.R., C.H., I.F., and T.I. designed the work. H.E.K., A.H.R., C.H., P.S., and T.I. performed the research. H.E.K., A.H.R., C.H., P.S., I.F., O.S., G.S.-R., and T.I. analyzed the data. H.E.K. and T.I. wrote the manuscript with contributions by the other authors. All authors critically read and revised the manuscript and approved the final version.

The author responsible for distribution of materials integral to the findings presented in this article in accordance with the policy described in the Instructions for Authors (https://academic.oup.com/plphys/pages/general-instructions) is Till Ischebeck (till.ischebeck@uni-muenster.de).

## References

[kiac127-B1] Allen JW , Tevatia R, Demirel Y, DiRusso CC, Black PN (2018) Induction of oil accumulation by heat stress is metabolically distinct from N stress in the green microalgae *Coccomyxa subellipsoidea* C169. PLoS ONE 13: 1–20 doi:10.1371/journal.pone.0204505 10.1371/journal.pone.0204505 PMC6160078 30261009

[kiac127-B2] Almeida J , Perez‐Fons L, Fraser PD (2021) A transcriptomic, metabolomic and cellular approach to the physiological adaptation of tomato fruit to high temperature. Plant Cell Environ 44: 2211–2229 doi:10.1111/pce 32691430 10.1111/pce.13854

[kiac127-B3] Beck JG , Mathieu D, Loudet C, Buchoux S, Dufourc EJ (2007) Plant sterols in “rafts”: a better way to regulate membrane thermal shocks. FASEB J 21: 1714–1723 doi 10.1096/fj.06-7809com 17317727 10.1096/fj.06-7809com

[kiac127-B4] Bellaire A , Ischebeck T, Staedler Y, Weinhaeuser I, Mair A, Parameswaran S, Ito T, Schönenberger J, Weckwerth W (2014) Metabolism and development - integration of micro computed tomography data and metabolite profiling reveals metabolic reprogramming from floral initiation to silique development. New Phytol 202: 322–335 doi 10.1111/nph.12631 24350948 10.1111/nph.12631 PMC4283998

[kiac127-B5] Berdyshev EV , Gorshkova IA, Garcia JGN, Natarajan V, Hubbard WC (2005) Quantitative analysis of sphingoid base-1-phosphates as bisacetylated derivatives by liquid chromatography–tandem mass spectrometry. Anal Biochem 339: 129–136 doi 10.1016/j.ab.2004.12.006 15766719 10.1016/j.ab.2004.12.006

[kiac127-B6] Berghoff SA , Spieth L, Sun T, Hosang L, Schlaphoff L, Depp C, Düking T, Winchenbach J, Neuber J, Ewers D, et al (2021) Microglia facilitate repair of demyelinated lesions via post-squalene sterol synthesis. Nat Neurosci 24: 47–60 doi 10.1038/s41593-020-00757-6 33349711 10.1038/s41593-020-00757-6 PMC7116742

[kiac127-B7] Boavida LC , McCormick S (2007) Temperature as a determinant factor for increased and reproducible *in vitro* pollen germination in *Arabidopsis thaliana*. Plant J 52: 570–582 doi:10.1111/j.1365-313X.2007.03248.x 17764500 10.1111/j.1365-313X.2007.03248.x

[kiac127-B8] Bonaventure G , Salas JJ, Pollard MR, Ohlrogge JB (2003) Disruption of the FATB gene in Arabidopsis demonstrates an essential role of saturated fatty acids in plant growth. Plant Cell 15: 1020–1033 doi:10.1105/tpc.008946 12671095 10.1105/tpc.008946 PMC152346

[kiac127-B9] Botte CY , Deligny M, Roccia A, Bonneau AL, Saïdani N, Hardré H, Aci S, Yamaryo-Botté Y, Jouhet J, Dubots E, et al (2011) Chemical inhibitors of monogalactosyldiacylglycerol synthases in *Arabidopsis thaliana*. Nat Chem Biol 7: 834–842 doi:10.1038/nchembio.658 21946275 10.1038/nchembio.658

[kiac127-B10] Chen J , Burke JJ, Xin Z, Xu C, Velten J (2006) Characterization of the Arabidopsis thermosensitive mutant *atts02* reveals an important role for galactolipids in thermotolerance. Plant Cell Environ 29: 1437–1448 doi:10.1111/j.1365-3040.2006.01527.x 17080965 10.1111/j.1365-3040.2006.01527.x

[kiac127-B11] Christenhusz MJM , Byng JW (2016) The number of known plants species in the world and its annual increase. Phytotaxa 261: 201–217 doi:10.11646/phytotaxa.261.3.1

[kiac127-B12] Coast O , Murdoch AJ, Ellis RH, Hay FR, Jagadish KSV (2016) Resilience of rice (*Oryza* spp.) pollen germination and tube growth to temperature stress. Plant Cell Environ 39: 26–37 doi:10.1111/pce.12475 25346255 10.1111/pce.12475

[kiac127-B13] Converti A , Casazza AA, Ortiz EY, Perego P, Del Borghi M (2009) Effect of temperature and nitrogen concentration on the growth and lipid content of *Nannochloropsis oculata* and *Chlorella vulgaris* for biodiesel production. Chem Eng Proc Process Intensificat 48: 1146–1151 doi:10.1016/j.cep.2009.03.006

[kiac127-B14] Demirel U , Morris WL, Ducreux LJM, Yavuz C, Asim A, Tindas I, Campbell R, Morris JA, Verrall SR, Hedley PE, et al (2020) Physiological, biochemical, and transcriptional responses to single and combined abiotic stress in stress-tolerant and stress-sensitive potato genotypes. Front Plant Sci 11: 1–21 doi:10.3389/fpls.2020.00169 32184796 10.3389/fpls.2020.00169 PMC7058966

[kiac127-B15] Deruyffelaere C , Purkrtova Z, Bouchez I, Collet B, Cacas JL, Chardot T, Gallois JL, D’Andrea S (2018) PUX10 is a CDC48A adaptor protein that regulates the extraction of ubiquitinated oleosins from seed lipid droplets in Arabidopsis. Plant Cell 30: 2116–2136 doi:10.1105/tpc.18.00275 30087208 10.1105/tpc.18.00275 PMC6181022

[kiac127-B16] Ding P , Rekhter D, Ding Y, Feussner K, Busta L, Haroth S, Xu S, Li X, Jetter R, Feussner I, et al (2016) Characterization of a pipecolic acid biosynthesis pathway required for systemic acquired resistance. Plant Cell 28: 2603–2615 doi:10.1105/tpc.16.00486 27758894 10.1105/tpc.16.00486 PMC5134984

[kiac127-B17] Dobin A , Davis CA, Schlesinger F, Drenkow J, Zaleski C, Jha S, Batut P, Chaisson M, Gingeras TR (2013) STAR: ultrafast universal RNA-seq aligner. Bioinformatics 29: 15–21 doi:10.1093/bioinformatics/bts635 23104886 10.1093/bioinformatics/bts635 PMC3530905

[kiac127-B18] Dufourc EJ (2008) Sterols and membrane dynamics. J Chem Biol 1: 63–77 doi 10.1007/s12154-008-0010-6 19568799 10.1007/s12154-008-0010-6 PMC2698314

[kiac127-B19] Edwards KD , Fernandez-Pozo N, Drake-Stowe K, Humphry M, Evans A, Bombarely A, Allen F, Hurst R, White B, Kernodle S, et al (2017) A reference genome for *Nicotiana tabacum* enables map-based cloning of homeologous loci implicated in nitrogen utilization efficiency. BMC Genom 18: 1–14 doi:10.1186/s12864-017-3791-6 10.1186/s12864-017-3791-6 PMC5474855 28625162

[kiac127-B20] Flores-Rentería L , Whipple AV, Benally GJ, Patterson A, Canyon B, Gehring CA (2018) Higher temperature at lower elevation sites fails to promote acclimation or adaptation to heat stress during pollen germination. Front Plant Sci 9: 1–14 doi:10.3389/fpls.2018.00536 29760715 10.3389/fpls.2018.00536 PMC5936790

[kiac127-B21] Fragkostefanakis S , Mesihovic A, Hu Y, Schleiff E (2016) Unfolded protein response in pollen development and heat stress tolerance. Plant Reprod 29: 81–91 doi:10.1007/s00497-016-0276-8 27022919 10.1007/s00497-016-0276-8

[kiac127-B22] Ghosh AK , Chauhan N, Rajakumari S, Daum G, Rajasekharan R (2009) AT4G24160, a soluble acyl-Coenzyme A-dependent lysophosphatidic acid acyltransferase. Plant Physiol 151: 869–881 doi:10.1104/pp.109.144261 19700561 10.1104/pp.109.144261 PMC2754629

[kiac127-B23] Han B , Yang N, Pu H, Wang T (2018) Quantitative proteomics and cytology of rice pollen sterol-rich membrane domains reveals pre-established cell polarity cues in mature pollen. J Proteome Res 17: 1532–1546 doi:10.1021/acs.jproteome.7b00852 29508613 10.1021/acs.jproteome.7b00852

[kiac127-B24] Haney CH , Long SR (2010) Plant flotillins are required for infection by nitrogen-fixing bacteria. Proc Natl Acad Sci USA 107: 478–483 doi:10.1073/pnas.0910081107 20018678 10.1073/pnas.0910081107 PMC2806772

[kiac127-B25] Harsh A , Sharma YK, Joshi U, Rampuria S, Singh G, Kumar S, Sharma R (2016) Effect of short-term heat stress on total sugars, proline and some antioxidant enzymes in moth bean (*Vigna aconitifolia*). Ann Agric Sci 61: 57–64 doi:10.1016/j.aoas.2016.02.001

[kiac127-B26] Härtel H , Dörmann P, Benning C (2000) DGD1-independent biosynthesis of extraplastidic galactolipids after phosphate deprivation in Arabidopsis. Proc Natl Acad Sci USA 97: 10649–10654 doi:10.1073/pnas.180320497 10973486 10.1073/pnas.180320497 PMC27079

[kiac127-B27] Hedhly A (2011) Sensitivity of flowering plant gametophytes to temperature fluctuations. Environ Exp Bot 74: 9–16 doi:10.1016/j.envexpbot.2011.03.016

[kiac127-B28] Henriksen JR , Andresen TL, Feldborg LN, Duelund L, Ipsen JH (2010) Understanding detergent effects on lipid membranes: a model study of lysolipids. Biophys J Biophys Soc 98: 2199–2205 doi:10.1016/j.bpj.2010.01.037 10.1016/j.bpj.2010.01.037 PMC2872274 20483328

[kiac127-B29] Hernández-Vega JC , Cady B, Kayanja G, Mauriello A, Cervantes N, Gillespie A, Lavia L, Trujillo J, Alkio M, Colón-Carmona A (2017) Detoxification of polycyclic aromatic hydrocarbons (PAHs) in *Arabidopsis thaliana* involves a putative flavonol synthase. J Hazard Mater 176: 139–148 doi:10.1016/j.jhazmat.2016.08.058 10.1016/j.jhazmat.2016.08.058 PMC5373802 27637093

[kiac127-B30] Herrfurth C , Liu YT, Feussner I (2021) Targeted analysis of the plant lipidome by UPLC-NanoESI-MS/MS. *In* Bartels D, Dörmann P, eds, Plant Lipids. Methods in Molecular Biology, Vol 2295. Humana, Louisville, KY, pp 135–155 doi:10.1007/978-1-0716-1362-7_9 10.1007/978-1-0716-1362-7_9 34047976

[kiac127-B31] Higashi Y , Okazaki Y, Myouga F, Shinozaki K, Saito K (2015) Landscape of the lipidome and transcriptome under heat stress in *Arabidopsis thaliana.* Sci Rep 5: 10533 doi:10.1038/srep10533 26013835 10.1038/srep10533 PMC4444972

[kiac127-B32] Higashi Y , Saito K (2019) Lipidomic studies of membrane glycerolipids in plant leaves under heat stress. Prog Lipid Res 75: 100990 doi:10.1016/j.plipres.2019.100990 31442527 10.1016/j.plipres.2019.100990

[kiac127-B33] Hornung E , Pernstich C, Feussner I (2002) Formation of conjugated Δ11 Δ13-double bonds by Δ12-linoleic acid (1,4)-acyl-lipid-desaturase in pomegranate seeds. Eur J Biochem 269: 4852–4859 doi:10.1046/j.1432-1033.2002.03184.x 12354116 10.1046/j.1432-1033.2002.03184.x

[kiac127-B34] Ischebeck T , Valledor L, Lyon D, Gingl S, Nagler M, Meijón M, Egelhofer V, Weckwerth W (2014) Comprehensive cell-specific protein analysis in early and late pollen development from diploid microsporocytes to pollen tube growth. Mol Cell Proteom 13: 295–310 doi:10.1074/mcp.M113.028100 10.1074/mcp.M113.028100 PMC3879621 24078888

[kiac127-B35] Ischebeck T (2016) Lipids in pollen—they are different. Biochim Biophys Acta 1861: 1315–1328 doi:10.1016/j.bbalip.2016.03.023 27033152 10.1016/j.bbalip.2016.03.023

[kiac127-B36] Ischebeck T , Krawczyk HE, Mullen RT, Dyer JM, Chapman KD (2020) Lipid droplets in plants and algae: distribution, formation, turnover and function. Semin Cell Dev Biol 108: 82–93. doi:10.1016/j.semcdb.2020.02.014 32147380

[kiac127-B37] Jacob P , Hirt H, Bendahmane A (2017) The heat-shock protein/chaperone network and multiple stress resistance. Plant Biotechnol J 15: 405–414 doi:10.1111/pbi.12659 27860233 10.1111/pbi.12659 PMC5362687

[kiac127-B38] James CN , Horn PK, Case CR, Gidda SK, Zhang D, Mullen RT, Dyer JM, Anderson RGW, Chapman KD (2010) Disruption of the Arabidopsis CGI-58 homologue produces Chanarin–Dorfman-like lipid droplet accumulation in plants. Proc Natl Acad Sci USA 107: 17833–17838 doi:10.1073/pnas.0911359107 20876112 10.1073/pnas.0911359107 PMC2955100

[kiac127-B39] Kaplan F , Kopka J, Haskell DW, Zhao W, Schiller KC, Gatzke N, Sung DY, Guy CL (2004) Exploring the temperature–stress metabolome. Plant Physiol 136: 4159–4168 doi:10.1104/pp.104.052142.1 15557093 10.1104/pp.104.052142 PMC535846

[kiac127-B40] Karapanos IC , Akoumianakis KA, Olympios CM, Passam HC (2010) Tomato pollen respiration in relation to *in vitro* germination and pollen tube growth under favourable and stress-inducing temperatures. Sex Plant Reprod 23: 219–224 doi:10.1007/s00497-009-0132-1 20063191 10.1007/s00497-009-0132-1

[kiac127-B41] Keller M, Hu Y , Mesihovic A, Fragkostefanakis S, Schleiff E, Simm S (2017) Alternative splicing in tomato pollen in response to heat stress. DNA Res 24: 205–217 doi 10.1093/dnares/dsw051 28025318 10.1093/dnares/dsw051 PMC5397606

[kiac127-B42] Keller M , Bokszczanin K, Bostan H, Bovy AG, Chen Y, Chaturvedi P, Chiusano M, Firon N, Fragkostefanakis S, Iannacone R, et al (2018) The coupling of transcriptome and proteome adaptation during development and heat stress response of tomato pollen. BMC Genom 19: 1–20 doi:10.1186/s12864-018-4824-5 10.1186/s12864-018-4824-5 PMC5994098 29884134

[kiac127-B43] Kelly A , Feussner I (2016) Oil is on the agenda: lipid turnover in higher plants. Biochim Biophys Acta 1861: 1253–1268 doi:10.1016/j.bbalip.2016.04.021 27155216 10.1016/j.bbalip.2016.04.021

[kiac127-B44] Kopka J , Schauer N, Krueger S, Birkemeyer C, Usadel B, Bergmüller E, Dörmann P, Weckwerth W, Gibon Y, Stitt M, et al (2005) GMD@CSB.DB: the golm metabolome database. Bioinformatics 21: 1635–1638 doi:10.1093/bioinformatics/bti236 15613389 10.1093/bioinformatics/bti236

[kiac127-B45] Kotak S , Larkindale J, Lee U, von Koskull-Döring P, Vierling E, Scharf KD (2007) Complexity of the heat stress response in plants. Curr Opin Plant Biol 10: 310–316 10.1016/j.pbi.2007.04.011 17482504

[kiac127-B46] Kretzschmar FK , Mengel LA, Müller AO, Schmitt K, Blersch KF, Valerius O, Braus GH, Ischebeck T (2018) PUX10 is a lipid droplet-localized scaffold protein that interacts with CELL DIVISION CYCLE48 and is involved in the degradation of lipid droplet proteins. Plant Cell 30: 2137–2160 doi:10.1105/tpc.18.00276 30087207 10.1105/tpc.18.00276 PMC6181012

[kiac127-B47] Kretzschmar FK , Doner NM, Krawczyk HE, Scholz P, Schmitt K, Valerius O, Braus GH, Mullen RT, Ischebeck T (2020) Identification of low-abundance lipid droplet proteins in seeds and seedlings. Plant Physiol 182: 1326–1345 doi:10.1104/pp.19.01255 31826923 10.1104/pp.19.01255 PMC7054876

[kiac127-B48] Lawas LMF , Li X, Erban A, Kopka J, Jagadish SVK, Zuther E, Hincha DK (2019) Metabolic responses of rice cultivars with different tolerance to combined drought and heat stress under field conditions. GigaScience 8: 1–21 doi:10.1093/gigascience/giz050 10.1093/gigascience/giz050 PMC6511916 31081890

[kiac127-B49] Lee JW , Nishiumi S, Yoshida M, Fukusaki E, Bamba T (2013) Simultaneous profiling of polar lipids by supercritical fluid chromatography/tandem mass spectrometry with methylation. J Chromatogr A 1279: 98–107 doi:10.1016/j.chroma.2013.01.020 23380365 10.1016/j.chroma.2013.01.020

[kiac127-B50] Légeret B , Schulz-Raffelt M, Nguyen HM, Auroy P, Beisson F, Peltier G, Blanc G, Li-Beisson Y (2016) Lipidomic and transcriptomic analyses of *Chlamydomonas reinhardtii* under heat stress unveil a direct route for the conversion of membrane lipids into storage lipids. Plant Cell Environ 39: 834–847 doi:10.1111/pce.12656 26477535 10.1111/pce.12656

[kiac127-B51] Li-Beisson Y , Shorrosh B, Beisson F, Andersson MX, Arondel V, Bates PD, Baud S, Bird D, DeBono A, Durrett TP (2013) Acyl-lipid metabolism. Arabidopsis Book 11: e0161 doi:10.1199/tab.0161 23505340 10.1199/tab.0161 PMC3563272

[kiac127-B52] Li F , Asami T, Wu X, Tsang EWT, Cutler AJ (2007) A putative hydroxysteroid dehydrogenase involved in regulating plant growth and development. Plant Physiol 145: 87–97 doi:10.1104/pp.107.100560 17616511 10.1104/pp.107.100560 PMC1976581

[kiac127-B53] Li R , Liu P, Wan Y, Chen T, Wang Q, Mettbach U, Baluska F, Samaj J, Fang X, Lucas WJ, et al (2012) A membrane microdomain-associated protein, Arabidopsis Flot1, is involved in a clathrin-independent endocytic pathway and is required for seedling development. Plant Cell 24: 2105–2122 doi:10.1105/tpc.112.095695 22589463 10.1105/tpc.112.095695 PMC3442590

[kiac127-B54] Liao Y , Smyth GK, Shi W (2014) FeatureCounts: an efficient general purpose program for assigning sequence reads to genomic features. Bioinformatics 30: 923–930 doi:10.1093/bioinformatics/btt656 24227677 10.1093/bioinformatics/btt656

[kiac127-B55] Lu J , Xu Y, Wang J, Singer SD, Chen G (2020) The role of triacylglycerol in plant stress response. Plants 9: 472 doi:10.3390/plants9040472 10.3390/plants9040472 PMC7238164 32276473

[kiac127-B56] Luria G , Rutley N, Lazar I, Harper JF, Miller G (2019) Direct analysis of pollen fitness by flow cytometry: implications for pollen response to stress. Plant J 98: 942–952 doi:10.1111/tpj.14286 30758085 10.1111/tpj.14286

[kiac127-B57] Luttgeharm KD , Kimberlin AN, Cahoon EB (2016) Plant sphingolipid metabolism and function. *In* Nakamura Y, Li-Beisson Y, eds, Lipids in Plant and Algae Development, Ed 86. Springer International Publishing, Berlin, Germany, pp 249–286 10.1007/978-3-319-25979-6_11 27023239

[kiac127-B58] Luttgeharm KD , Kimberlin AN, Cahoon RE, Cerny RL, Napier JA, Markham JE, Cahoon EB (2015) Sphingolipid metabolism is strikingly different between pollen and leaf in Arabidopsis as revealed by compositional and gene expression profiling. Phytochemistry 115: 121–129 doi:10.1016/j.phytochem.2015.02.019 25794895 10.1016/j.phytochem.2015.02.019

[kiac127-B59] Mamode Cassim A , Gouguet P, Gronnier J, Laurent N, Germain V, Grison M, Boutté Y, Gerbeau-Pissot P, Simon-Plas F, Mongrand S (2019) Plant lipids: key players of plasma membrane organization and function. Prog Lipid Res 73: 1–27 doi:10.1016/j.plipres.2018.11.002 30465788 10.1016/j.plipres.2018.11.002

[kiac127-B60] Markham JE , Li J, Cahoon EB, Jaworski JG (2006) Separation and identification of major plant sphingolipid classes from leaves. J Biol Chem 281: 22684–22694 doi:10.1074/jbc.M604050200 16772288 10.1074/jbc.M604050200

[kiac127-B61] Mascarenshas JP (1993) Molecular mechanisms of pollen tube growth and differentiation. Plant Cell 5: 1303–1314 doi: 10.1105/tpc.5.10.1303 12271030 PMC160363

[kiac127-B62] Matyash V , Liebisch G, Kurzchalia TV, Shevchenko A, Schwudke D (2008) Lipid extraction by methyl-tert-butyl ether for high-throughput lipidomics. J Lipid Res 49: 1137–1146 doi:10.1194/jlr.D700041-JLR200 18281723 10.1194/jlr.D700041-JLR200 PMC2311442

[kiac127-B63] McCarthy DJ , Chen Y, Smyth GK (2012) Differential expression analysis of multifactor RNA-Seq experiments with respect to biological variation. Nucleic Acids Res 40: 4288–4297 doi:10.1093/nar/gks042 22287627 10.1093/nar/gks042 PMC3378882

[kiac127-B64] Michaelson LV , Napier JA, Molino D, Faure JD (2016) Plant sphingolipids: their importance in cellular organization and adaption. Biochim Biophys Acta Mol Cell Biol Lipids 1861: 1329–1335 doi:10.1016/j.bbalip.2016.04.003 10.1016/j.bbalip.2016.04.003 PMC4970446 27086144

[kiac127-B65] Mittal D , Madhyastha DA, Grover A (2012) Genome-wide transcriptional profiles during temperature and oxidative stress reveal coordinated expression patterns and overlapping regulons in rice. PLoS ONE 7: e40899 doi:10.1371/journal.pone.0040899 22815860 10.1371/journal.pone.0040899 PMC3397947

[kiac127-B66] Moreno-Pérez AJ , Venegas-Calerón M, Vaistij FE, Salas JJ, Larson TR, Garcés R, Graham IA, Martínez-Force E (2012) Reduced expression of FatA thioesterases in Arabidopsis affects the oil content and fatty acid composition of the seeds. Planta 235: 629–639 doi:10.1007/s00425-011-1534-5 22002626 10.1007/s00425-011-1534-5

[kiac127-B67] Mueller SP , Krause DM, Mueller MJ, Fekete A (2015) Accumulation of extra-chloroplastic triacylglycerols in Arabidopsis seedlings during heat acclimation. J Exp Bot 66: 4517–4526 doi:10.1093/jxb/erv226 25977236 10.1093/jxb/erv226 PMC4507766

[kiac127-B68] Mueller SP , Unger M, Guender L, Fekete A, Mueller MJ (2017) Phospholipid: diacylglycerol acyltransferase-mediated triacylglyerol synthesis augments basal thermotolerance. Plant Physiol 175: 486–497 doi:10.1104/pp.17.00861 28733391 10.1104/pp.17.00861 PMC5580778

[kiac127-B69] Muhlemann JK , Younts TLB, Muday GK (2018) Flavonols control pollen tube growth and integrity by regulating ROS homeostasis during high-temperature stress. Proc Natl Acad Sci USA 115: E11188–E11197 doi:10.1073/pnas.1811492115 30413622 10.1073/pnas.1811492115 PMC6255205

[kiac127-B70] Müller AO , Ischebeck T (2018) Characterization of the enzymatic activity and physiological function of the lipid droplet-associated triacylglycerol lipase AtOBL1. New Phytol 217: 1062–1076 doi:10.1111/nph.14902 29178188 10.1111/nph.14902

[kiac127-B71] Müller F , Rieu I (2016) Acclimation to high temperature during pollen development. Plant Reprod 29: 107–118 doi:10.1007/s00497-016-0282-x 27067439 10.1007/s00497-016-0282-x PMC4909792

[kiac127-B72] Nakamura Y , Kobayashi K, Ohta H (2009) Activation of galactolipid biosynthesis in development of pistils and pollen tubes. Plant Physiol Biochem 47: 535–539 doi:10.1016/j.plaphy.2008.12.018 19181535 10.1016/j.plaphy.2008.12.018

[kiac127-B73] Návarová H , Bernsdorff F, Döring AC, Zeier J (2013) Pipecolic acid, an endogenous mediator of defense amplification and priming, is a critical regulator of inducible plant immunity. Plant Cell 24: 5123–5141 doi:10.1105/tpc.112.103564 10.1105/tpc.112.103564 PMC3556979 23221596

[kiac127-B74] Niu Y , Xiang Y (2018) An overview of biomembrane functions in plant responses to high-temperature stress. Front Plant Sci 9: 1–18 doi:10.3389/fpls.2018.00915 30018629 10.3389/fpls.2018.00915 PMC6037897

[kiac127-B75] Pérez-Rodríguez P , Riaño-Pachón DM, Guedes Corrêa LG, Rensing SA, Kersten B, Mueller-Roeber B (2010) PlnTFDB: updated content and new features of the plant transcription factor database. Nucleic Acids Res 38: D822–D827 doi:10.1093/nar/gkp805 19858103 10.1093/nar/gkp805 PMC2808933

[kiac127-B76] Pidkowich MS , Nguyen HT, Heilmann I, Ischebeck T, Shanklin J (2007) Modulating seed -ketoacyl-acyl carrier protein synthase II level converts the composition of a temperate seed oil to that of a palm-like tropical oil. Proc Natl Acad Sci USA 104: 4742–4747 doi:10.1073/pnas.0611141104 17360594 10.1073/pnas.0611141104 PMC1838670

[kiac127-B77] Platre MP , Bayle V, Armengot L, Bareille J, Marquès-Bueno MDM, Creff A, Maneta-Peyret L, Fiche JB, Nollmann M, Miège C, et al (2019) Developmental control of plant Rho GTPase nano-organization by the lipid phosphatidylserine. Science 364: 57–62 doi:10.1126/science.aav9959 30948546 10.1126/science.aav9959

[kiac127-B78] Popko J , Herrfurth C, Feussner K, Ischebeck T, Iven T, Haslam R, Hamilton M, Sayanova O, Napier J, Khozin-Goldberg I, et al (2016) Metabolome analysis reveals betaine lipids as major source for triglyceride formation, and the accumulation of sedoheptulose during nitrogen-starvation of *Phaeodactylum tricornutum*. PLoS ONE 11: 1–23 doi:10.1371/journal.pone.0164673 10.1371/journal.pone.0164673 PMC5063337 27736949

[kiac127-B79] Priya M , Dhanker OP, Siddique Kadambot HM, HanumanthaRao B, Nair Ramakrishnan M, Pandey S, Singh S, Varshney RK, Prasad PV, Nayyar H (2019) Drought and heat stress-related proteins: an update about their functional relevance in imparting stress tolerance in agricultural crops. Theor Appl Genet 132: 1607–1638 doi:10.1007/s00122-019-03331-2 30941464 10.1007/s00122-019-03331-2

[kiac127-B80] Puli MR , Rajsheel P, Aswani V, Agurla S, Kuchitsu K, Raghavendra AS (2016) Stomatal closure induced by phytosphingosine-1-phosphate and sphingosine-1-phosphate depends on nitric oxide and pH of guard cells in *Pisum sativum.* Planta 244: 831–841 doi:10.1007/s00425-016-2545-z 27233507 10.1007/s00425-016-2545-z

[kiac127-B81] Rahmati Ishka M , Brown E, Weigand C, Tillett RL, Schlauch KA, Miller G, Harper JF (2018) A comparison of heat-stress transcriptome changes between wild-type Arabidopsis pollen and a heat-sensitive mutant harboring a knockout of cyclic nucleotide-gated cation channel 16 (*cngc16*). BMC Genom 19: 1–19 doi:10.1186/s12864-018-4930-4 10.1186/s12864-018-4930-4 PMC6057101 30041596

[kiac127-B82] Raja MM , Vijayalakshmi G, Naik ML, Basha PO, Sergeant K, Hausman JF, Sha Valli Khan PS (2019) Pollen development and function under heat stress: from effects to responses. Acta Physiol Plant 41: 47 doi:10.1007/s11738-019-2835-8

[kiac127-B83] Read SM , Clarke AE, Bacic A (1993) Stimulation of growth of cultured *Nicotiana tabacum* W38 pollen tubes by poly(ethylene glycol) and Cu(II) salts. Protoplasma 177: 1–14 doi:10.1007/BF01403393

[kiac127-B84] Robinson MD , McCarthy DJ, Smyth GK (2009) edgeR: a bioconductor package for differential expression analysis of digital gene expression data. Bioinformatics 26: 139–140 doi:10.1093/bioinformatics/btp616 19910308 10.1093/bioinformatics/btp616 PMC2796818

[kiac127-B85] Rotsch AH , Kopka J, Feussner I, Ischebeck T (2017) Central metabolite and sterol profiling divides tobacco male gametophyte development and pollen tube growth into eight metabolic phases. Plant J 92: 129–146 doi:10.1111/tpj.13633 28685881 10.1111/tpj.13633

[kiac127-B86] Santiago JP , Sharkey TD (2019) Pollen development at high temperature and role of carbon and nitrogen metabolites. Plant Cell Environ 42: 2759–2775 doi:10.1111/pce.13576 31077385 10.1111/pce.13576

[kiac127-B87] Scholz P , Anstatt J, Krawczyk HE, Ischebeck T (2020) Signalling pinpointed to the tip: the complex regulatory network that allows pollen tube growth. Plants 9: 1098 doi:10.3390/plants9091098 10.3390/plants9091098 PMC7569787 32859043

[kiac127-B88] Shao Q , Liu X, Su T, Ma C, Wang P (2019) New insights into the role of seed oil body proteins in metabolism and plant development. Front Plant Sci 10: 1–14 doi:10.3389/fpls.2019.01568 31921234 10.3389/fpls.2019.01568 PMC6914826

[kiac127-B89] Shi W , Li X, Schmidt RC, Struik PC, Yin X, Jagadish SVK (2018) Pollen germination and *in vivo* fertilization in response to high-temperature during flowering in hybrid and inbred rice. Plant Cell Environ 41: 1287–1297 doi:10.1111/pce.13146 29336039 10.1111/pce.13146

[kiac127-B90] Shiva S , Samarakoon T, Lowe KA, Roach C, Vu HS, Colter M, Porras H, Hwang C, Roth MR, Tamura P, et al (2020) Leaf lipid alterations in response to heat stress of *Arabidopsis thaliana.* Plants 9: 845 doi:10.3390/plants9070845 10.3390/plants9070845 PMC7412450 32635518

[kiac127-B91] Simon-Plas F , Perraki A, Bayer E, Gerbeau-Pissot P, Mongran S (2011) An update on plant membrane rafts. Curr Opin Plant Biol 14: 642–649 doi:10.1016/j.pbi.2011.08.003 21903451 10.1016/j.pbi.2011.08.003

[kiac127-B92] Simons K , Ikonen E (1997) Functional rafts in cell membranes. Nature 387: 569–572 doi:10.1038/42408 9177342 10.1038/42408

[kiac127-B93] Snider JL , Oosterhuis DM, Loka DA, Kawakami EM (2011a) High temperature limits *in vivo* pollen tube growth rates by altering diurnal carbohydrate balance in field-grown *Gossypium hirsutum* pistils. J Plant Physiol 168: 1168–1175 doi:10.1016/j.jplph.2010.12.011 21256621 10.1016/j.jplph.2010.12.011

[kiac127-B94] Snider JL , Oosterhuis DM, Kawakami EM (2011b) Diurnal pollen tube growth rate is slowed by high temperature in field-grown *Gossypium hirsutum* pistils. J Plant Physiol 168: 441–448 doi:10.1016/j.jplph.2010.08.003 20832140 10.1016/j.jplph.2010.08.003

[kiac127-B95] Song G , Wang M, Zeng B, Zhang J, Jiang C, Hu Q, Geng G, Tang C (2015) Anther response to high-temperature stress during development and pollen thermotolerance heterosis as revealed by pollen tube growth and *in vitro* pollen vigor analysis in upland cotton. Planta 241: 1271–1285 doi:10.1007/s00425-015-2259-7 25672505 10.1007/s00425-015-2259-7

[kiac127-B96] Spicher L , Glauser G, Kessler F (2016) Lipid antioxidant and galactolipid remodeling under temperature stress in tomato plants. Front Plant Sci 7: 1–12 doi:10.3389/fpls.2016.00167 26925083 10.3389/fpls.2016.00167 PMC4756161

[kiac127-B97] Sprunck S (2020) Twice the fun, double the trouble: gamete interactions in flowering plants. Curr Opin Plant Biol 53: 106–116 doi:10.1016/j.pbi.2019.11.003 31841779 10.1016/j.pbi.2019.11.003

[kiac127-B98] Staff IA , Taylor PE, Kenrick J, Knox RB (1989) Ultrastructural analysis of plastids in angiosperm pollen tubes. Sex Plant Reprod 2: 70–76 doi:10.1007/BF00191993

[kiac127-B99] Sunoj VSJ , Somayanda IM, Chiluwal A, Perumal R, Vara Prasad PV, Jagadish KSV (2017) Resilience of pollen and post-flowering response in diverse sorghum genotypes exposed to heat stress under field conditions. Crop Sci 57: 1658–1669 doi:10.2135/cropsci2016.08.0706

[kiac127-B100] Touraine B , Vignols F, Przybyla-Toscano J, Ischebeck T, Dhalleine T, Wu HC, Magno C, Berger N, Couturier J, Dubos C, et al (2019) Iron–sulfur protein NFU2 is required for branched-chain amino acid synthesis in Arabidopsis roots. J Exp Bot 70: 1875–1889 doi:10.1093/jxb/erz050 30785184 10.1093/jxb/erz050

[kiac127-B101] Villette C , Berna A, Compagnon V, Schaller H (2015) Plant sterol diversity in pollen from angiosperms. Lipids 50: 749–760 doi:10.1007/s11745-015-4008-x 25820807 10.1007/s11745-015-4008-x

[kiac127-B102] Wu J , James DW, Jr, Dooner HK, Browse J (1994) A mutant of Arabidopsis deficient in the elongation of palmitic acid. Plant Physiol 106: 143–150 doi:10.1104/pp.106.1.143 12232312 10.1104/pp.106.1.143 PMC159509

[kiac127-B103] Yang H , Yang S, Li Y, Hua J (2007) The Arabidopsis BAP1 and BAP2 genes are general inhibitors of programmed cell death. Plant Physiol 145: 135–146 doi:10.1104/pp.107.100800 17631528 10.1104/pp.107.100800 PMC1976577

[kiac127-B104] Yang Y , Mininberg B, Tarbet A, Weathers P (2013) At high temperature lipid production in *Ettlia oleoabundans* occurs before nitrate depletion. Appl Microbiol Biotechnol 97: 2263–2273 doi:10.1007/s00253-012-4671-2 23334512 10.1007/s00253-012-4671-2

[kiac127-B105] Zhang C , Li G, Chen T, Feng B, Fu W, Yan J, Islam MR, Jin Q, Tao L, Fu G (2018) Heat stress induces spikelet sterility in rice at anthesis through inhibition of pollen tube elongation interfering with auxin homeostasis in pollinated pistils. Rice 11: 14 doi:10.1186/s12284-018-0206-5 29532187 10.1186/s12284-018-0206-5 PMC5847639

[kiac127-B106] Zhang Z , Cheng ZJ, Gan L, Zhang H, Wu FQ, Lin QB, Wang JL, Wang J, Guo XP, Zhang X, et al (2016) OsHSD1, a hydroxysteroid dehydrogenase, is involved in cuticle formation and lipid homeostasis in rice. Plant Sci 249: 35–45 doi:10.1016/j.plantsci.2016.05.005 27297988 10.1016/j.plantsci.2016.05.005

[kiac127-B107] Zhao Q , Zhou L, Liu J, Du X, Asad MAU, Huang F, Pan G, Cheng F (2018) Relationship of ROS accumulation and superoxide dismutase isozymes in developing anther with floret fertility of rice under heat stress. Plant Physiol Biochem 122: 90–101 doi:10.1016/j.plaphy.2017.11.009 29202329 10.1016/j.plaphy.2017.11.009

[kiac127-B108] Zinta G , AbdElgawad H, Peshev D, Weedon JT, Van den Ende W, Nijs I, Janssens IA, Beemster GTS, Asard H (2018) Dynamics of metabolic responses to periods of combined heat and drought in *Arabidopsis thaliana* under ambient and elevated atmospheric CO_2_. J Exp Bot 69: 2159–2170 doi:10.1093/jxb/ery055 29462345 10.1093/jxb/ery055 PMC6019062

