# Supplementary material for: Heat stress leads to rapid lipid remodeling and transcriptional adaptations in Nicotiana tabacum pollen tubes
Source: Plant Physiol. 2022 Mar 18;189(2):490–515. doi: 10.1093/plphys/kiac127 (PMC9157110; doi:10.1093/plphys/kiac127)
Supplement: kiac127_Supplementary_Data [file kiac127_supplementary_data.zip › Supplemental table S1.pdf]

Supplemental Table S1 Parameters for lipid analysis by UPLC-nanoESI-MS/MS

**Lipid Category Class**	**Subclass**	**Solvent start condition [% B]**	**Flow rate [ml/min]**	**Ionization mode**	**Q1**	**Q3**	**DP [V]**	**EP [V]**	**CE [V]**	**CXP [V]**
**Glycerolipids**										
Galactolipids	DGDG	65	0.1	Negative	$[M+OAc]^-$	$[RCOO_{sn1}]^-$ / $[RCOO_{sn2}]^-$	-100	-10	-40	-10
	DGMG	40	0.1	Negative	$[M+OAc]^-$	$[RCOO]^-$	-200	-10	-40	-11
	MGDG	65	0.1	Negative	$[M+OAc]^-$	$[RCOO_{sn1}]^-$ / $[RCOO_{sn2}]^-$	-100	-10	-45	-10
	MGMG	40	0.1	Negative	$[M+OAc]^-$	$[RCOO]^-$	-200	-10	-40	-11
	SQDG	65	0.1	Negative	$[M-H]^-$	$[RCOO_{sn1}]^-$ / $[RCOO_{sn2}]^-$	-100	-10	-40	-10
	SQMG	40	0.1	Negative	$[M-H]^-$	$[RCOO]^-$	-200	-10	-40	-11
Neutral lipids	DAG	80	0.1	Positive	$[M+NH_4]^+$	$[M-RCOO]^+$	100	10	38	10
	TAG	90	0.13	Positive	$[M+NH_4]^+$	$[M-RCOO]^+$	140	10	40	6
Phospholipids	LPA	40	0.1	Negative[a]	$[M+Me-H]^-$	$[RCOO]^-$	-200	-10	-30	-11
	LPC	40	0.1	Negative	$[M+OAc]^-$	$[RCOO]^-$	-200	-10	-40	-11
	LPE	40	0.1	Negative	$[M-H]^-$	$[RCOO]^-$	-200	-10	-40	-11
	LPG	40	0.1	Negative	$[M-H]^-$	$[RCOO]^-$	-200	-10	-40	-11
	LPI	40	0.1	Negative	$[M-H]^-$	$[RCOO]^-$	-200	-10	-40	-11
	LPS	40	0.1	Negative	$[M-H]^-$	$[RCOO]^-$	-200	-10	-40	-11
	PA	65	0.1	Negative[a]	$[M+Me-H]^-$	$[RCOO_{sn1}]^-$ / $[RCOO_{sn2}]^-$	-200	-10	-38	-11
	PC	65	0.1	Negative	$[M+OAc]^-$	$[RCOO_{sn1}]^-$ / $[RCOO_{sn2}]^-$	-100	-10	-40	-10
	PE	65	0.1	Negative	$[M-H]^-$	$[RCOO_{sn1}]^-$ / $[RCOO_{sn2}]^-$	-100	-10	-40	-10
	PG	65	0.1	Negative	$[M-H]^-$	$[RCOO_{sn1}]^-$ / $[RCOO_{sn2}]^-$	-100	-10	-40	-10
	PI	65	0.1	Negative	$[M-H]^-$	$[RCOO_{sn1}]^-$ / $[RCOO_{sn2}]^-$	-100	-10	-40	-10
	PIP	65	0.1	Negative[a]	$[M+Me-H]^-$	$[RCOO_{sn1}]^-$ / $[RCOO_{sn2}]^-$	-200	-10	-60	-11
	PIP_2	65	0.1	Negative[a]	$[M+Me-H]^-$	$[RCOO_{sn1}]^-$ / $[RCOO_{sn2}]^-$	-200	-10	-60	-11
	PS	65	0.1	Negative	$[M-H]^-$	$[RCOO_{sn1}]^-$ / $[RCOO_{sn2}]^-$	-100	-10	-40	-10
**Sphingolipids**										
	LCB	40	0.1	Positive	$[M+H]^+$	$[M-H_2O+H]^+$ / $[M-2H_2O+H]^+$ / $[M-3H_2O+H]^+$	50	10	20/ 25/ 30	10

Neutral lipids	Cer	65	0.1	Positive	$[M+H]^+$	$[LCB-2H_2O+H]^+$	100	10	50	10
Glycolipids	HexCer	65	0.1	Positive	$[M+H]^+$	$[LCB-2H_2O+H]^+$	120	10	55	10
	IPC<br>GlcA-IPC	65	0.1	Positive	$[M+H]^+$	$[Cer-2H_2O+H]^+$	180	10	40	10
	Hex-GlcA-IPC<br>HexNAc-GlcA-IPC<br>Hex-Hex-GlcA-IPC<br>Hex-HexNAc-GlcA-IPC	65	0.1	Positive	$[M+NH_4]^+$	$[Cer-2H_2O+H]^+$	160	10	76	10
Phospholipids	Cer-P	65	0.1	Negative[b]	$[M+2Ac-H]^-$	$[M+Ac-H]^-$ / $[M-H_2O-H]^-$	-100	-10	-50	-10
	LCB-P	40	0.1	Negative[b]	$[M+3Ac-H]^-$ & $[M+2Ac-H]^-$	$[M+2Ac-H]^-$ / $[M+Ac-H_2O-H]^-$ & $[M+Ac-H]^-$ / $[M-H_2O-H]^-$	-100	-10	-31	-10
**Sterol lipids**										
Glycolipids	SG	65	0.1	Positive	$[M+NH_4]^+$	$[Sterol-OH]^+$	100	10	22	10
	ASG	65	0.1	Positive	$[M+NH_4]^+$	$[Sterol-OH]^+$	100	10	28	10
Neutral lipids	SE	90	0.13	Positive	$[M+NH_4]^+$	$[Sterol-OH]^+$	140	10	22	6

The ionization mode depicts the polarity of the nanoESI source. Q1 and Q3 indicate the parent and product ions, respectively. CP, EP, CE and CXP indicate the declustering potential, entrance potential, collision energy and cell exit potential for the molecular species of the corresponding lipid classes, respectively.

Abbreviations for lipid classes and subclasses: ASG, acylated steryl glycoside; Cer, ceramide; Cer-P, ceramide phosphate; DAG, diacylglycerol; DGDG, digalactosyldiacylglycerol; DGMG, digalactosylmonoacylglycerol; GlcA, glucuronosyl; Hex, hexosyl; HexCer, hexosylceramide; HexN, hexosaminyl; HexNAc, *N*-acetylhexosaminyl; IPC, inositol phosphoceramide; LCB, long chain base; LCB-P, long chain base phosphate; LPA, lysophosphatidic acid; LPC, lysophosphatidylcholine; LPE, lysophosphatidylethanolamine; LPG, lysophosphatidylglycerol; LPI, lysophosphatidylinositol; LPS, lysophosphatidylserine; PA, phosphatidic acid; PC, phosphatidylcholine; PE, phosphatidylethanolamine; PG, phosphatidylglycerol; PI, phosphatidylinositol; PIP, phosphatidylinositol phosphate; PIP_2, phosphatidylinositol bisphosphate; PS, phosphatidylserine; MGDG, monogalactosyldiacylglycerol; MGMG, monogalactosylmonoacylglycerol; SE, steryl ester; SG, steryl glycoside; SQDG, sulfoquinovosyldiacylglycerol; SQMG, sulfoquinovosylmonoacylglycerol; TAG, triacylglycerol.

[a]Methylation before UPLC-nanoESI-MS/MS analysis; [b]Acetylation before UPLC-nanoESI-MS/MS analysis.
